# Three Decades of Use of the Minimum Basic Data Set in Infectious Disease Research in Spain: A Scoping Review with an Evidence-Mapping Approach

**DOI:** 10.3390/tropicalmed11020061

**Published:** 2026-02-20

**Authors:** Beatriz Rodríguez-Alonso, Hugo Almeida, Montserrat Alonso-Sardón, Inmaculada Izquierdo, Ángela Romero-Alegría, Amparo López-Bernús, Virginia Velasco-Tirado, Josué Pendones Ulerio, Javier Pardo Lledías, Moncef Belhassen-García

**Affiliations:** 1 Servicio de Medicina Interna, Unidad de Enfermedades Infecciosas, Hospital Universitario de Salamanca, 37007 Salamanca, Spain; beamedicina@gmail.com (B.R.-A.); ivh.inmaizquierdo@gmail.com (I.I.); aralegria@yahoo.es (Á.R.-A.); alopezb@saludcastillayleon.es (A.L.-B.); belhassen@usal.es (M.B.-G.); 2 Centro de Investigación de Enfermedades Tropicales de la Universidad de Salamanca (CIETUS), 37007 Salamanca, Spain; sardonm@usal.es (M.A.-S.); virvela@yahoo.es (V.V.-T.); jpendones@saludcastillayleon.es (J.P.U.); 3 Instituto de Investigación Biomédica de Salamanca (IBSAL), 37007 Salamanca, Spain; 4 Área de Medicina Preventiva, Epidemiología y Salud Pública, Facultad de Medicina, Universidad de Salamanca, 37007 Salamanca, Spain; 5 Servicio de Dermatología, Hospital Universitario de Salamanca, 37007 Salamanca, Spain; 6 Servicio de Microbiología y Parasitología, Hospital Universitario de Salamanca, 37007 Salamanca, Spain; 7 Servicio de Medicina Interna, Hospital Marqués de Valdecilla, 39008 Santander, Spain; javier.pardo@scsalud.es; 8 Instituto de Investigación Valdecilla (IDIVAL), 39008 Santander, Spain

**Keywords:** Minimum Basic Data Set (CMBD), infectious diseases, hospital discharge database, administrative health data, scoping review, epidemiology

## Abstract

Nationwide hospital discharge databases are increasingly used in infectious disease research, yet their methodological strengths and limitations are rarely synthesised. In Spain, the Minimum Basic Data Set (Conjunto Mínimo Básico de Datos, CMBD) was implemented in 1987 and provides near-universal coverage of acute-care hospitalisations and has been widely applied in infectious disease epidemiology. However, its overall contribution and intrinsic constraints have not been comprehensively mapped. Given the breadth of infections, study designs, populations and outcome definitions in CMBD-based research, effect-size synthesis was not feasible; therefore, we conducted a scoping review with an evidence-mapping approach. We aimed to synthesise the scope, applications and methodological limitations of CMBD-based infectious disease research since its implementation. We conducted a scoping review following JBI guidance and reported according to PRISMA-ScR. PubMed, Embase, Web of Science and Scopus were searched from inception to 25 November 2024 for peer-reviewed journal articles in English or Spanish using CMBD data to investigate infectious diseases in Spain (no restrictions were applied by study design; grey literature was excluded). Screening, data charting and synthesis were completed during 2025. Four reviewers independently screened records and charted data. Studies were classified by infectious disease focus, syndromic category, study design and geographical scope. A total of 359 studies published between 1996 and 2024 were included, mostly retrospective observational analyses. Infectious diseases were the primary focus in 225 studies (62.7%), most commonly respiratory, gastrointestinal/liver and vaccine-preventable infections. Subnational analyses were concentrated in a limited number of regions. Over 80% of reported limitations reflected intrinsic CMBD features. Over three decades, the CMBD has become a cornerstone of hospital-based infectious disease research in Spain, enabling robust national analyses. However, limitations in clinical detail, microbiological confirmation and coding consistency constrain aetiological specificity and causal inference, highlighting the need for data validation and linkage with complementary sources.

## 1. Introduction

Nationwide hospital discharge databases are increasingly used in infectious disease research to estimate hospital burden, monitor temporal trends and evaluate the impact of public health interventions at scale. However, the methodological implications of relying on administrative coding systems, particularly in the infectious disease field, remain insufficiently synthesised.

In Spain, the Conjunto Mínimo Básico de Datos (CMBD) is a mandatory nationwide hospital discharge database that currently captures more than 95% of acute-care admissions, encompassing both public and private hospitals. It was approved by the Interterritorial Council of the National Health System (SNS) in 1987 and implemented as a nationally coordinated hospital discharge administrative dataset within the SNS. Its coverage was progressively extended, with incorporation of private-sector hospital discharge data starting in 2005. Thus, CMBD provides nationwide (state-level) information on the great majority of acute-care hospital discharges. In 2016, it evolved into the RAE-CMBD model, extending the registry beyond inpatient hospitalisation and consolidating coverage of private-sector activity [1,2,3]. For simplicity, we use “CMBD” as an umbrella term for the registry across its evolution; when referring specifically to the extended model implemented from 2016 onwards, we use “RAE-CMBD”. Its longitudinal depth and near-universal coverage make it a potentially powerful tool for population-level infectious disease epidemiology [3,4,5,6]. The CMBD has been progressively refined, including the addition of funding variables (1998), the incorporation of patient identifiers and clinical service designation (2005), and transition from ICD-9-CM to ICD-10-ES coding (2016) [7]. It captures standardised information on diagnoses (ICD-coded), procedures, age, sex, hospital, length of stay and discharge type, among other variables, supporting research, surveillance and healthcare planning [8,9,10,11].

The Conjunto Mínimo Básico de Datos (CMBD) data have been widely used to investigate infectious diseases, including vaccine-preventable infections, respiratory diseases, sepsis, healthcare-associated infections and emerging or neglected pathogens. These studies have addressed diverse research questions, ranging from temporal trends and geographical patterns to hospital outcomes, costs and the impact of vaccination and other public health interventions [8,9,10], and to estimate incidence, evaluate interventions, track emerging/re-emerging infections, and describe geographical and demographic patterns [8,9,10,11]. However, ICD-code validity can vary—particularly for infections with complex diagnostic criteria or low specificity—limiting aetiological and outcome assessments [12,13]. While ICD-10-ES improves coding granularity, it complicates longitudinal comparability, reinforcing the need for periodic validation and robust methods [12,13,14,15]. Given the substantial burden of infectious diseases in Spain (notably respiratory infections, sepsis and healthcare-associated infections) [16], nationwide administrative data sources such as the CMBD remain crucial for monitoring trends, assessing vaccination programmes and supporting responses to public health emergencies, including the COVID-19 pandemic [8,11,16].

In parallel, bibliometric studies indicate that Spanish scientific output in Infectious Diseases and Microbiology has achieved sustained growth and strong international visibility. For 2000–2013, Spain ranked among the leading countries worldwide in both categories, and a more recent update for 2014–2021 identified high levels of international collaboration and a substantial proportion of publications in first-quartile journals, underscoring the maturity and impact of the Spanish research ecosystem in these fields [17].

Despite its widespread use, persistent concerns about coding quality, the lack of microbiological variables and the absence of post-discharge follow-up underscore the need for scoping reviews and evidence-mapping analyses aimed at synthesising and systematising the use of CMBD data in infectious disease research [8,11,12,13].

Regardless of more than three decades of CMBD-based infectious disease research, no previous review has systematically mapped how this database has been used, nor critically appraised its methodological strengths and intrinsic limitations. Given the breadth of infections, study designs, populations, coding eras and outcome definitions in CMBD-based research, effect-size synthesis is not feasible or informative. Accordingly, we conducted a scoping review with an evidence-mapping approach to describe the research landscape, summarise commonly assessed outcome domains and author-reported limitations, and identify gaps that could inform future-focused systematic reviews.

## 2. Materials and Methods

### 2.1. Protocol

**Figure 1:**
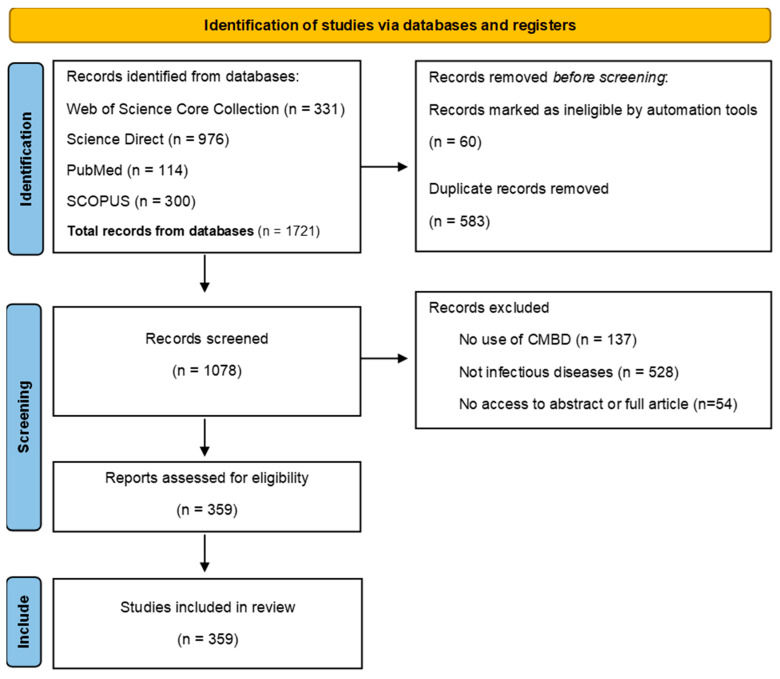
PRISMA-ScR flow diagram. Adapted from Tricco et al. (2018) [18].

The review was conducted and reported in accordance with the PRISMA-ScR reporting guideline for scoping reviews [18] and informed by JBI methodological guidance [19]. Elements from PRISMA 2020 and relevant sections of the Cochrane Handbook were used where applicable [20,21]. The PRISMA-ScR flow diagram summarising the selection process is provided in Figure 1. The review methods (eligibility criteria, screening process and data charting items) were defined a priori in an internal protocol.

#### 2.1.1. Eligibility Criteria

Consistent with scoping review methodology, we applied broad inclusion criteria to encompass the full range of CMBD-based infectious disease research, regardless of study design or outcome measures. We included sources of evidence that: (i) were published as peer-reviewed journal articles in English or Spanish; (ii) explicitly employed the CMBD as a primary or combined data source; (iii) examined infectious diseases or infectious complications as primary or secondary outcomes; and (iv) were conducted in, or analysed data originating from, Spain. No restrictions on publication date were imposed. We excluded studies that did not use the CMBD, focused exclusively on non-infectious conditions, were considered grey literature (including abstracts, dissertations, editorials and news reports) or for which the full text could not be obtained.

#### 2.1.2. Information Sources and Search Strategy

A comprehensive search strategy was designed to identify all relevant studies on the use of the Minimum Basic Data Set (CMBD) for the study of infectious diseases in Spain. Searches were conducted across the following databases: Web of Science Core Collection, PubMed, Scopus and Science Direct. The last search was carried out on 25 November 2024. Screening, data charting and synthesis were conducted during 2025 due to the large number of eligible studies and the need for duplicate review and consensus procedures. This evidence map represents a snapshot of the literature up to 25 November 2024 and is intended to be updated periodically (e.g., every 5 years) or after major CMBD structural changes. The search combined both controlled vocabulary terms (e.g., Medical Subject Headings [MeSH] or Emtree terms) and free-text keywords. Terms related to the CMBD (“Minimum Basic Data Set,” “Minimum Basic Dataset,” “Conjunto Mínimo Básico de Datos,” CMBD, MBDS) were paired with terms describing infectious diseases (“infectious diseases,” “enfermedades infecciosas,” “infection,” “infección”) and limited to studies conducted in or referring to Spain (España). Boolean operators were used to combine these terms appropriately across databases.

Search strategies were tailored to each database’s syntax and vocabulary (MeSH/Emtree terms and free-text keywords). In PubMed, we combined CMBD-related terms with an infection concept using controlled vocabulary (MeSH) and free-text terms. For other sources with platform-specific syntax constraints (e.g., limited support for truncation/wildcards), we used a broader CMBD and Spain query to maximise sensitivity. To remain consistent with the infection concept applied in PubMed, we implemented a two-stage selection approach for records retrieved from these broader searches. In stage 1, we screened titles/abstracts to identify studies using CMBD in Spain. In stage 2, we verified infectious disease relevance by checking for infection-related terminology in searchable fields (title, abstract and keywords) using terms aligned with the PubMed infection concept (e.g., infection, infectious, “infec*” and closely related terms). Only studies meeting the predefined infectious disease eligibility criteria were included.

The complete database-specific search strategies exactly as executed are provided in Supplementary File S2: Search strategy.

#### 2.1.3. Study Selection

All retrieved references were imported into a reference-management software (Rayyan^®^) http://rayyan.qcri.org (accessed on 13 November 2025). Duplicates were identified and removed both automatically and manually.

After deduplication, titles and abstracts were screened independently by four reviewers to identify potentially relevant articles. The full texts of all records considered potentially eligible were subsequently assessed in detail. Discrepancies at any stage were resolved through discussion and, when necessary, consultation with a senior reviewer.

#### 2.1.4. Data Charting

Following JBI guidance for scoping reviews, we developed and pilot-tested a standardised data charting form on 10 randomly selected studies. Four reviewers (BRA, HA, MAS and MBG) independently charted data in parallel, with disagreements resolved by consensus. Data items included: author and publication year, journal, study design, type of infectious disease or condition studied, study objective and period of the study, geographic scope (national, regional, or hospital-specific), primary versus secondary outcome classification, and funding status. In addition, we also charted the main reported outcome domain(s), and the stated scientific contribution/significance as described by the authors. These items were coded into non-mutually exclusive categories (a study could contribute to more than one category); therefore, percentages do not sum to 100%. Outcomes were mapped at the domain level due to heterogeneity in definitions, populations, ICD coding eras and analytic approaches; no pooling of effect estimates was attempted. Additional methodological information such as sample size, statistical methods, and coding system version (ICD-9-CM or ICD-10-ES) was recorded when available. Limitations were extracted only when explicitly stated by the study authors (author-reported). We did not infer additional limitations beyond those reported. Author-reported limitations were coded into predefined domains by two reviewers, with disagreements resolved by consensus.

Consistent with the exploratory aims of this scoping review, we systematically charted information on methodological limitations and data quality concerns reported in each study. Given the substantial heterogeneity in reported outcomes and analytic approaches, we did not attempt to tabulate or pool quantitative results across studies, focusing instead on cataloguing the types of limitations described by authors, with particular attention to those stemming from inherent properties of the CMBD database (for example, restrictions on clinical severity data, absence of laboratory confirmations, lack of post-discharge follow-up, coding inconsistencies across ICD eras, and missing or incomplete variables). Items not reported in the original articles were coded as “n.r.” (not reported), and no imputation was performed.

#### 2.1.5. Data Synthesis

Given the anticipated clinical and methodological heterogeneity across infectious conditions, populations, time periods, and outcome definitions, we did not plan a formal meta-analysis or quantitative pooling of effect estimates. Instead, we conducted a narrative synthesis summarising the evolution of CMBD-based infectious disease research over time, the range of infections and research questions addressed, the principal uses of CMBD data (including surveillance, burden-of-disease estimation, vaccine evaluation, environmental determinants, and health-services research), and recurrent methodological strengths and limitations, with particular attention to those stemming from structural features of the CMBD itself. Study characteristics and thematic domains were summarised using descriptive statistics (counts, proportions, and trends over time).

#### 2.1.6. Risk of Bias Assessment

The primary aim of this review was to map and describe how the CMBD has been used for infectious disease research, rather than to synthesise effect estimates from a set of comparable analytic studies. Consistent with current guidance for scoping reviews, we did not conduct a formal risk of bias assessment of individual studies using standard tools such as the Newcastle–Ottawa Scale or ROBINS-I. These instruments are designed for specific observational designs (e.g., cohort or case–control studies) and focus on the internal validity of exposure–outcome associations; they are less applicable to the heterogeneous body of evidence included here, which encompasses descriptive time trend analyses, ecological studies, methodological validations, economic evaluations and registry-linkage studies. Applying a single checklist across such diverse designs would likely yield misleading global scores and obscure the structural sources of bias common to CMBD-based research.

Instead, we adopted an evidence-mapping approach. For each included study, we extracted and summarised information on potential sources of bias and methodological limitations, whether explicitly reported by the authors or inferred from the description of methods, with particular attention to domains commonly considered in observational research (selection of the study population, comparability of groups where applicable, and ascertainment and coding of infectious outcomes). These aspects are synthesised qualitatively in the Section 3 and Section 4, with a focus on data quality and completeness, representativeness, and the potential for residual and unmeasured confounding inherent to administrative hospital discharge databases. In keeping with the mapping objectives of this scoping review, this approach enables us to characterise recurrent strengths and weaknesses of CMBD-based infectious disease studies without assigning numerical quality scores that are not comparable across designs.

### 2.2. Study Design and Research Question

We framed our study question using the Population–Concept–Context (PCC) framework recommended for scoping reviews. The population of interest comprised patients whose hospitalisations are captured in the Spanish CMBD. The core concept was the use of CMBD data for infectious disease research (including epidemiology, burden of disease, vaccine evaluations, environmental determinants and infection-related complications). The context was the Spanish hospital system, including national and regional CMBD data.

### 2.3. Classification of Infectious Disease Focus (Primary vs. Secondary)

For the purposes of this review, we classified the role of infectious diseases in each study as either primary or secondary. A study was deemed to have a primary infectious focus when its main objective explicitly centred on one or more infectious diseases (such as their epidemiology, outcomes, costs, or the impact of vaccines or targeted treatments) or when the primary outcome/main endpoint was itself an infectious event, for instance sepsis, postsurgical infection, pneumonia, or an infectious complication arising from another underlying condition. Conversely, we considered a study to have a secondary infectious focus when infectious outcomes were examined only within a broader set of endpoints (e.g., complications of chronic diseases, hospital performance indicators, or safety metrics), or when infections were included merely as covariates, non-significant variables, or secondary outcomes without constituting the core of the research question. Two reviewers applied this classification independently. Disagreements were infrequent and resolved by consensus; when uncertainty persisted, studies were conservatively classified as having a secondary infectious focus.

## 3. Results

### 3.1. General Characteristics

**Figure 2:**
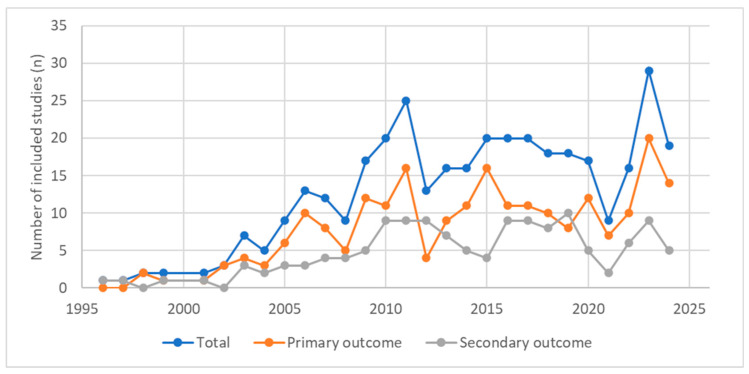
Publication trends over time.

We identified 359 articles that met the inclusion criteria and were included in the evidence map of CMBD-based infectious disease research. Full details are provided in Supplementary Tables S1 and S2 (Refs. [22,23,24,25,26,27,28,29,30,31,32,33,34,35,36,37,38,39,40,41,42,43,44,45,46,47,48,49,50,51,52,53,54,55,56,57,58,59,60,61,62,63,64,65,66,67,68,69,70,71,72,73,74,75,76,77,78,79,80,81,82,83,84,85,86,87,88,89,90,91,92,93,94,95,96,97,98,99,100,101,102,103,104,105,106,107,108,109,110,111,112,113,114,115,116,117,118,119,120,121,122,123,124,125,126,127,128,129,130,131,132,133,134,135,136,137,138,139,140,141,142,143,144,145,146,147,148,149,150,151,152,153,154,155,156,157,158,159,160,161,162,163,164,165,166,167,168,169,170,171,172,173,174,175,176,177,178,179,180,181,182,183,184,185,186,187,188,189,190,191,192,193,194,195,196,197,198,199,200,201,202,203,204,205,206,207,208,209,210,211,212,213,214,215,216,217,218,219,220,221,222,223,224,225,226,227,228,229,230,231,232,233,234,235,236,237,238,239,240,241,242,243,244,245,246,247,248,249,250,251,252,253,254,255,256,257,258,259,260,261,262,263,264,265,266,267,268,269,270,271,272,273,274,275,276,277,278,279,280,281,282,283,284,285,286,287,288,289,290,291,292,293,294,295,296,297,298,299,300,301,302,303,304,305,306,307,308,309,310,311,312,313,314,315,316,317,318,319,320,321,322,323,324,325,326,327,328,329,330,331,332,333,334,335,336,337,338,339,340,341,342,343,344,345,346,347,348,349,350,351,352,353,354,355,356,357,358,359,360,361,362,363,364,365,366,367,368,369,370,371,372,373,374,375,376]). The first eligible CMBD-based infectious disease study was published in 1996. Publication output increased markedly over time: 6 studies in the 1990s, 77 in the 2000s, 186 in the 2010s and 90 between 2020 and 2024. Overall, the studies analysed CMBD data covering hospitalisations from 1971 to 2023, with median study periods centred around the early-mid 2000s (Tables S1 and S2). Publication trends over time are shown in Figure 2.

Most studies were retrospective observational (Table 1).

Funding information was poorly reported (no funding statement in 74.9% of articles), particularly in older studies. Only 90 studies (25.1%) explicitly declared external funding.

### 3.2. Primary Versus Secondary Infection Outcomes

Primary-focus studies included those in which (i) the main objective was the description or analysis of one or more infectious diseases, or (ii) the primary endpoint was an infectious event (e.g., sepsis, pneumonia, nosocomial infection). Secondary-focus studies were those in which infections appeared only as part of a wider set of outcomes (e.g., complications of chronic conditions, performance indicators or safety outcomes) or as non-significant covariates.

The proportion of primary infectious studies increased over time. In the 1990s, 3 out of 6 publications (50.0%) had a primary infectious focus; this proportion rose to 52/77 (67.5%) in 2000–2009, 107/186 (57.5%) in 2010–2019 and 63/90 (70.0%) from 2020 onwards.

Among the 225 studies with a primary infectious focus, the conditions most frequently investigated were vaccine-preventable and respiratory infections. When aggregating closely related topics, rotavirus gastroenteritis and vaccination were evaluated in 19 studies, while pneumococcal disease (including invasive pneumococcal disease and pneumococcal pneumonia) featured in 17 studies. *Bordetella pertussis* was the focus of 10 studies, and varicella/chickenpox and herpes zoster together accounted for approximately 20 analyses. Tuberculosis was examined in 10 studies, often in relation to temporal trends, comorbidities or clinical outcomes. Seasonal influenza was considered in eight studies and respiratory syncytial virus (RSV) in four. Finally, COVID-19/SARS-CoV-2 and its complications (including bacterial and fungal coinfections and coinfections with viral hepatitis) were explicitly addressed in around 16 primary-focus studies.

When grouped into syndromic categories, respiratory infections other than COVID-19 remained the most frequent primary-focus area (61/225; 27.1%), followed by gastrointestinal and liver infections (35/225; 15.6%) and multiple/broad infectious categories (25/225; 11.1%) (Table 2). Other prominent categories included other vaccine-preventable infections (21/225; 9.3%), invasive bacterial and CNS infections (17/225; 7.6%), COVID-19 (16/225; 7.1%), imported/tropical and parasitic infections (14/225; 6.2%), and HIV and sexually transmitted infections (13/225; 5.8%). Less frequently represented were healthcare-associated and postsurgical infections (11/225; 4.9%), other specific infections (10/225; 4.4%), and urinary tract infections (2/225; 0.9%).

The 134 secondary-focus studies predominantly analysed infections as complications or safety outcomes in non-infectious conditions. Typical examples included hospitalisations for cardiovascular disease, chronic obstructive pulmonary disease, cancer, hip fracture or frailty, where infections were captured as postoperative complications, causes of readmission or contributors to mortality. In other articles, infections were one of several variables in assessments of hospital efficiency, quality-of-care indicators or utilisation patterns.

### 3.3. Study Objectives and Outcome Domains

To strengthen the evidence-mapping deliverable, we summarised the most frequent study objective domains, outcome domains and stated scientific contributions (Table 3).

The most reported outcome domains were incidence, rates and burden (236/359, 65.7%), mortality (120/359, 33.4%), complications and adverse events (69/359, 19.2%), economic outcomes (48/359, 13.4%), time trends (47/359, 13.1%), length of stay (30/359, 8.4%) and risk factor and association analyses (28/359, 7.8%). As categories are non-mutually exclusive, a single study could contribute to multiple domains.

### 3.4. Geographical Distribution Within Spain

**Figure 3:**
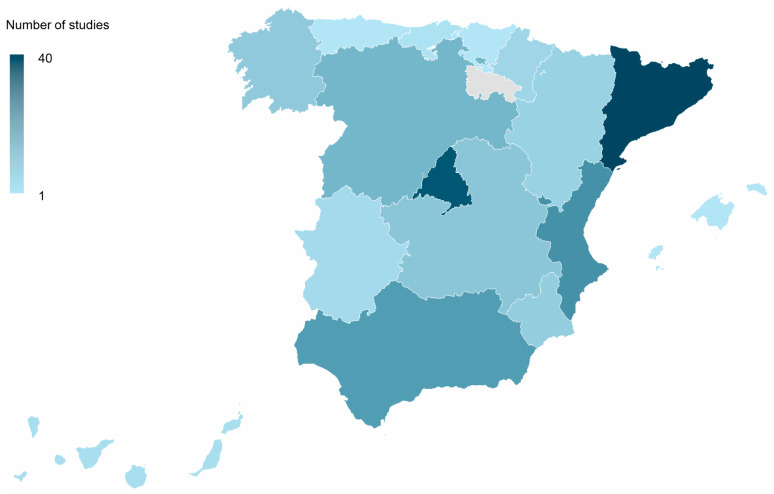
Geographical distribution of CMBD-based infectious disease studies by autonomous community, Spain (1996–2024). These figures reflect the geographic focus of individual analyses and do not necessarily correspond to the distribution of CMBD coverage itself.

Subnational studies showed a heterogeneous territorial distribution. According to the regional summary (Figure 3), Catalonia and the Community of Madrid were the most frequently represented autonomous communities, contributing 40 and 36 studies, respectively, followed by the Valencian Community (22) and Andalusia (19). Castilla y León (13), Castilla La Mancha (9), Galicia (8) and the Region of Murcia (7) also featured prominently. In contrast, some regions such as the Basque Country, Asturias and the Balearic Islands were only sporadically represented. These figures reflect the geographic focus of individual analyses and do not necessarily correspond to the distribution of CMBD coverage itself.

### 3.5. Methodological Limitations

This study approach prioritised feasibility and broad sensitivity; however, some diagnosis-specific CMBD studies may be under-represented if infection-related terminology is not captured in indexing or searchable fields.

This evidence map should be interpreted considering the intrinsic constraints of administrative discharge data. Across studies, author-reported limitations most frequently related to coding variability or misclassification (31.8%), lack of microbiology or pathogen confirmation (22.6%), and data quality, incomplete capture or heterogeneity (21.7%) (Table S3). The “Other/unclear” category captured non-specific limitation statements that were not reliably assignable to a single domain. These constraints do not invalidate CMBD research, but delimit the types of inferences that can be made (e.g., limited pathogen-level attribution, potential misclassification and residual confounding), and they highlight where linkage to microbiology or vaccination registries and methodological standardisation could most improve future research.

## 4. Discussion

In this scoping review with an evidence-mapping approach, we identified 359 CMBD-based studies on infectious diseases in Spain. Most studies addressed epidemiology or burden (198/359, 55.2%) and mortality, outcomes or severity (92/359, 25.6%), and the main reported results included incidence, rates or burden (236/359, 65.7%) and mortality (120/359, 33.4%) (Table 3). Evidence was concentrated in several syndromic areas (Table 2). Author-reported limitations predominantly reflected intrinsic constraints of administrative discharge data, especially coding variability or misclassification (31.8%), lack of microbiology or pathogen confirmation (22.6%) and data quality or incomplete capture (21.7%) (Table S3). We show a marked increase in the use of the CMBD over time, with infectious diseases playing an increasingly central role in national and regional analyses. Approximately two thirds of the studies treated infections as the primary focus of investigation, and the remainder considered them as secondary outcomes or safety indicators within broader clinical or health-services research questions, highlighting that the CMBD is no longer used solely as an administrative registry but has progressively become a key resource for epidemiology and health-services research [377,378]. However, the considerable heterogeneity in study designs, populations, time periods and outcome definitions precludes formal meta-analysis and limits the extent to which firm causal conclusions can be drawn from the mapped body of evidence.

From a methodological standpoint, an important advantage of the CMBD is that it is a national hospital discharge registry, implying mandatory and near-universal coverage and thereby avoiding the handicap of under-reporting that is inherent to conventional notifiable disease systems or sentinel surveillance networks [1,2,379]. This makes it particularly valuable for estimating the burden of hospitalisation and describing temporal and geographical patterns of severe infections [377,378]. At the same time, our synthesis highlights several intrinsic structural limitations of the CMBD. The database does not routinely capture detailed clinical variables such as severity scores or vital signs, laboratory or microbiological confirmation, vaccination status or post-discharge outcomes. More than four out of five limitations identified in the included studies were directly attributable to these inherent features of the CMBD rather than to study-specific analytic choices. These intrinsic limitations systematically constrain aetiological specificity and render the CMBD unsuitable for individual-level causal inference. Nevertheless, they do not diminish its value for estimating hospital burden, describing temporal and geographical trends, and evaluating the impact of large-scale public health interventions. In addition, CMBD-based research is inherently subject to coding-related bias. Diagnoses and procedures are assigned by trained administrative coders rather than by clinicians, and despite standardised training and periodic updates, misclassification and inter-centre variability remain unavoidable sources of uncertainty that must be considered when interpreting CMBD-based infectious disease estimates [380,381,382]. Similar strengths and limitations have been described for other national hospital discharge databases, including Hospital Episode Statistics in the United Kingdom and the Programme de *Médicalisation des Systèmes d’Information* in France. This consistency suggests that the methodological challenges identified in CMBD-based infectious disease research are not unique to Spain and that the lessons derived from this review are broadly transferable to other settings relying on administrative hospital data [381,382,383].

In this context, our findings support prioritising the strengthening and optimisation of the CMBD, including its expanded RAE-CMBD framework, rather than investing in new parallel notification systems that are likely to be more vulnerable to under-reporting and selection biases. Improving coding quality, validating diagnostic algorithms and enhancing data linkage are likely to yield greater benefits for infectious disease surveillance and research than the development of narrower, disease-specific registries [1,2]. Incorporating a carefully selected set of clinical, microbiological and treatment-related variables into the CMBD, together with improved linkage to microbiology, immunisation and notifiable disease registries, would substantially enhance its value for infectious disease research and surveillance [379,381]. Despite the limitations outlined above, the CMBD remains the most robust and comprehensive data source currently available in Spain for estimating the hospital and economic impact of infectious diseases, enabling detailed analyses of resource use, length of stay and direct hospital costs at a national scale [2,379]. Investing in better coding quality, targeted validation studies and the progressive enrichment of CMBD data fields is therefore likely to yield greater benefits than building new, narrower registries, and would consolidate the CMBD as a central pillar of a modern, data-driven system for infectious disease surveillance and health-policy evaluation.

This scoping review has limitations inherent to its methodology: The search was restricted to the sources consulted and the predefined eligibility criteria, and therefore relevant studies may have been missed (e.g., grey literature or non-indexed publications). In addition, consistent with the aim of evidence mapping, no formal appraisal of methodological quality or risk of bias of the included studies was conducted; consequently, the findings should be interpreted as descriptive. As with any evidence map, our findings reflect the literature up to the last search date (25 November 2024) and may require periodic updating.

## 5. Conclusions

We mapped 359 CMBD-based infectious disease studies in Spain and observed increasing publication activity over time. Most studies focused on incidence/burden and mortality outcomes. Author-reported limitations mainly reflected intrinsic constraints of administrative discharge data, particularly limited clinical granularity, lack of microbiological confirmation and coding variability. This evidence map highlights research concentrations and gaps and supports prioritisation of future-focused research and registry enhancements.

## Figures and Tables

**Table 1:** Main characteristics of studies.

Characteristic	Category	n = 359	% of All Studies
Study design	Retrospective (non-cohort)	229	63.8
	Retrospective cohort	19	5.3
	Cross-sectional	27	7.5
	Prospective observational	8	2.2
	Ecological/time-series/case-crossover	14	3.9
	Review/consensus	9	2.5
	Other designs	53	14.8
Geographical scope	National/multicentre	180	50.1
	Regional/provincial/hospital	176	49
	International	1	0.3
	Not reported	2	0.6
Infection outcome	Primary infectious outcome	225	62.7
	Secondary infectious outcome	134	37.3
Funding	Funded	90	25.1
	Funding not declared	269	74.9

**Table 2:** Infectious disease categories among CMBD-based studies with a primary infectious focus.

Category	Included Studies Condition	n (%)	Evidence Level
Respiratory infections (non-COVID-19)	Pneumococcal infection; *Bordetella pertussis*; Community-acquired pneumonia; Tuberculosis; Influenza	61 (27.1%)	High
Gastrointestinal and liver infections	Rotavirus (virus and vaccines); *Clostridium difficile* infection; Hepatitis C virus infection	35 (15.6%)	High
COVID-19	SARS-CoV-2 and associated infections	16 (7.1%)	Moderate
HIV and sexually transmitted infections	HIV infection; *Human papillomavirus* (vaccine, infection, associated cancer)	13 (5.8%)	Moderate
Invasive bacterial, sepsis and central nervous system infections	Invasive pneumococcal disease (IPD); Meningococcal infection; Infective endocarditis; Sepsis;	17 (7.6%)	Moderate
Healthcare-associated and postsurgical infections	Surgical site infection; Postoperative infection/sepsis and other complications; Nosocomial bacteremia; Rotavirus (nosocomial infection)	11 (4.9%)	Moderate
Urinary tract infections	Urinary tract infection (UTI) and associated comorbidities	2 (0.9%)	Low
Tropical and parasitic infections	Bartonella henselae; Rickettsia conorii (Mediterranean spotted fever); *Echinococcus granulosus*; *Leishmaniasis*; Malaria	14 (6.2%)	Moderate
Other vaccine-preventable infections	Herpes zoster infection (Chickenpox/varicella); Postherpetic neuralgia;	21 (9.3%)	High
Multiple broad infectious categories	Ambulatory care sensitive conditions; All-cause emergency case-mix	25 (11.1%)	High
Other specific infection-related conditions	Hemophagocytic syndrome (HPS); Diabetic foot; Malignant external otitis; Mucormycosis	10 (4.4%)	Moderate

Thresholds used: high ≥20 studies; moderate 6–19; low ≤5.

**Table 3:** Evidence map: objectives, outcomes, and contribution.

Category	n (%)
Study objective	
Epidemiology, burden (incidence, prevalence, rates)	198 (55.2)
Mortality, outcomes, severity	92 (25.6)
Trends over time	61 (17.0)
Economic, cost analysis	50 (13.9)
Quality, performance evaluation	28 (7.8)
Risk factors, determinants	21 (5.8)
Surveillance, monitoring, validation, methods	19 (5.3)
Main results	
Incidence, rates, burden	236 (65.7)
Mortality	120 (33.4)
Complications, adverse events	69 (19.2)
Costs, economic outcomes	48 (13.4)
Trends (increase, decrease)	47 (13.1)
Length of stay	30 (8.4)
Risk factors, associations	28 (7.8)
Scientific Contribution/Significance	
CMBD utility, value for research, surveillance	232 (64.6)
National-level evidence	72 (20.1)
Surveillance, monitoring implications	56 (15.6)
Validation, methodological contribution	29 (8.1)
Novel contribution (“*First-of-its-kind*”)	27 (7.5)
Economic burden relevance	21 (5.8)
Policy, planning, stakeholder relevance	14 (3.9)

Categories are non-mutually exclusive; a single study may contribute to more than one category. Percentages therefore do not sum to 100%.

## Data Availability

The study-level extracted dataset supporting the findings is available in Supplementary Material.

## Supplementary Materials

The following supporting information can be downloaded at: https://www.mdpi.com/article/10.3390/tropicalmed11020061/s1, Supplementary File S1: Preferred Reporting Items for Systematic reviews and Meta-Analyses extension for Scoping Reviews (PRISMA-ScR) Checklist; Supplementary File S2: Search strategy; Table S1: Studies included and characteristics. Infections as a primary outcome or result; Table S2: Studies included and characteristics. Infections as a secondary outcome or result; Table S3: Limitations domains and frequency.

## Abbreviations

The following abbreviations are used in this manuscript:

CMBD	*Conjunto Mínimo Básico de Datos*
COVID-19	Coronavirus disease 2019
HIV	Human immunodeficiency virus
HPV	Human papillomavirus
ICD	International Classification of Diseases
MBDS	Minimum Basic Data Set
n.r.	Not reported
PCC	Population–Concept–Context
RAE-CMBD	Expanded CMBD framework (enhanced Spanish hospital discharge database)
ROBINS-I	Risk of bias in non-randomised studies of interventions
RSV	Respiratory syncytial virus
SARS-CoV-2	Severe acute respiratory syndrome coronavirus 2
