# Supplementary material for: Three Decades of Use of the Minimum Basic Data Set in Infectious Disease Research in Spain: A Scoping Review with an Evidence-Mapping Approach
Source: Trop Med Infect Dis. 2026 Feb 20;11(2):61. doi: 10.3390/tropicalmed11020061 (PMC12945255; doi:10.3390/tropicalmed11020061)
Supplement: Supplementary file 1 [file tropicalmed-11-00061-s001.zip › Supplementary File S2. Search strategy v2.pdf]

Supplementary File S2. Search strategy.

Executed 25 November 2024.

General: No limits/filters applied.

Exported on: 25 November 2024.

A) **PubMed**("Minimum Basic Data Set" OR "Minimum Basic Dataset" OR "Conjunto Minimo Basico de Datos" OR CMBD OR MBDS) AND ("Infection" OR infect* OR "infectious disease*" OR "infectious diseases" OR "enfermedades infecciosas" OR infecci*) AND (Spain OR Espana OR España).

B) Scopus
TITLE-ABS-KEY("minimum basic data set" OR "minimum basic dataset" OR "conjunto minimo basico de datos" OR CMBD OR MBDS) AND TITLE-ABS-KEY(spain OR espana OR spanish)

C) Web of Science Core Collection
TS=("minimum basic data set" OR "minimum basic dataset" OR "conjunto minimo basico de datos" OR CMBD OR MBDS) AND TS=(spain OR espana OR spanish)

D) Science Direct
("Minimum Basic dataset" OR "Conjunto Minimo Basico de Datos") AND (Spain OR Espana OR España).
