# Supplementary material for: Three Decades of Use of the Minimum Basic Data Set in Infectious Disease Research in Spain: A Scoping Review with an Evidence-Mapping Approach
Source: Trop Med Infect Dis. 2026 Feb 20;11(2):61. doi: 10.3390/tropicalmed11020061 (PMC12945255; doi:10.3390/tropicalmed11020061)
Supplement: Supplementary file 1 [file tropicalmed-11-00061-s001.zip › Table S1. Infections as a primary outcome or result.pdf]

Table S1. Studies included and characteristics. Infections as a primary outcome or result.

Study ID		*Main Findings*		
Author (year) [Ref.]	*Journal*	*Condition studied*	*Study Objective*	*Study region (and period)*
Aguiló et al. (2005) **[22]**	*Gestión de Riesgos*	Postoperative infection and other complications	Describe postoperative adverse events and associated risk factors in general surgery.	Valencia (n.a.)
Aguiló et al. (2005) **[23]**	*Cirugía Española*	Acute appendicitis	Describe adverse outcomes after appendectomy and analyse associated factors.	Valencia (1994-1998)
Aldaz et al. (2007) **[24]**	*Enfermedades Infecciosas y Microbiología Clínica*	HIV	To describe trends in mortality and causes of death among HIV-positive individuals.	Navarra (1985-2004)
Almeida et al. (2023) **[25]**	*PLoS ONE*	*Babesiosis*	*To analyse the epidemiological impact of inpatients diagnosed with babesiosis*	*National (1997-2019)*
Alonso-Fernández et al. (2015) **[26]**	*Revista de Calidad Asistencial*	Clostridium difficile infection (CDI)	To evaluate concordance between microbiological diagnosis and discharge coding of CDI.	Madrid (2013)
Álvarez-del Río et al. (2023) **[27]**	*Frontiers in Public Health*	SARS-COV-2	To estimate prevalence and cost of hospitalisations with asymptomatic COVID-19 in Spain.	National (2020)
oÁlvaro-Meca et al. (2011) **[28]**	*Pediatric Infectious Disease Journal*	HIV infected children and Cancer	To evaluate trends in cancer incidence among HIV-infected children during HAART era.	National (1997-2008)
Álvaro-Meca et al. (2013) **[29]**	*Cancer Epidemiology*	Postoperative complications	To model long-term health transitions and complications following pancreatic cancer surgery.	National (1999-2009)
Álvaro-Meca et al. (2013) **[30]**	*BMC Infectious Diseases*	Candidiasis	To estimate rates of candidiasis among HIV-infected children using CMBD data.	National (1997-2008)
Álvaro-Meca et al. (2014) **[31]**	*Journal of Infection*	Tuberculosis (TB) and HIV coinfection	To estimate incidence, recurrence, and mortality of TB by HIV status in Spain.	National (1997-2010)
Álvaro-Meca et al. (2015) **[32]**	*HIV Medicine*	Nontuberculous mycobacterial infections (NTM)	To estimate incidence and mortality of NTM disease and trends by HIV status.	National (1997-2010)
Álvaro-Meca et al. (2016) **[33]**	*BMC Infectious Diseases*	Chronic hepatitis C	To analyse the impact of chronic hepatitis C (CHC) on mortality of cirrhotic ICU patients according to severe sepsis and cirrhosis stage.	National (2005-2010)
Álvaro-Meca et al. (2018) **[34]**	*Population Health Metrics*	Sepsis	To analyse national trends in incidence, mortality, and costs of sepsis hospitalisations in Spain.	National (2000-2013)
Álvaro-Meca et al. (2018) **[35]**	*Scientific Reports*	HIV and Pulmonary embolism (PE)	To evaluate PE-related hospitalisation and mortality trends among people living with HIV.	National (1997-2013)
Álvaro-Meca et al. (2018) **[36]**	*European Journal of Internal Medicine*	HIV/HCV-coinfected patients and deep venous thrombosis (DVT)	To analyse trends of DVT hospitalisations in HIV-infected patients and association with HCV coinfection.	National (1997-2013)
Álvaro-Meca et al. (2020) **[37]**	*Journal of Clinical Medicine*	Hepatitis C and sepsis	To analyse epidemiological trends of sepsis-related admissions, deaths, and costs in chronic hepatitis C patients.	National (2000-2015)
Álvaro-Meca et al. (2020) **[38]**	*Environmental Research*	Sepsis-related pneumonia	To evaluate the impact of short-term exposure to environmental factors on hospital admissions for sepsis-related pneumonia in Spain.	National (2013)
Álvaro-Meca et al. (2022) **[39]**	*Environmental Research*	Acute viral lower respiratory tract infections (LRTI)	To evaluate association between short-term environmental exposure	National (2013-2015)

			and acute LRTI hospitalisations in children <2 years.	
Álvaro-Meca et al.(2016) **[40]**	*PLoS ONE*	Tuberculosis (TB) and HIV coinfection	To evaluate environmental determinants of TB-related hospitalisations in HIV-positive individuals.	National (1997-2012)
Álvaro-Meca et al. (2022) **[41]**	*Environmental Health: A Global Access Science Source*	Viral lower respiratory tract infection (LRTI)	To assess environmental factors associated with viral LRTI hospitalisations in older adults.	National (2013-2015)
Anegón Blanco et al. (2006) **[42]**	*Medicina Clínica*	Malaria	To describe hospitalisations due to malaria and estimate incidence and costs in Spain.	National (1999-2002)
Ángeles Gutiérrez Rodríguez et al. (2008) **[43]**	*Medicina Clínica*	Pneumococcal disease	To describe magnitude and evolution of pneumococcal disease in Madrid before and after vaccine introduction.	Madrid (1998-2006)
Aranaz et al. (2003) **[44]**	*Cirugía Española*	Surgical site infections (nosocomial)	To analyse adverse events in general and digestive surgery.	Alicante (1996-2000)
Ares-Gómez et al. (2024) **[45]**	*The Lancet Infectious Diseases*	Respiratory syncytial virus (RSV)	To assess effectiveness of universal nirsevimab prophylaxis against RSV-related hospitalisations.	Galicia (2016-2023)
Arias-Fernández et al. (2021) **[46]**	*Vaccine*	Pneumonia	To assess the burden of pneumonia with viral-bacterial coinfection among older adults during six influenza seasons.	National (2009-2015)
Ariza-Mejía et al. (2010) **[47]**	*Enfermeria Clinica*	Gonococcal infection	To estimate hospitalisation rates for gonococcal infection in Madrid.	Madrid (1997-2006)
Arteaga et al. (2009) **[48]**	*Journal of Viral Hepatitis*	Hepatitis A virus infection	To analyse hospitalisations, comorbidities, and costs of hepatitis A.	National (2000-2005)
Arteaga-Rodríguez et al. (2010) **[49]**	*European Journal of Gastroenterology and Hepatology*	Acute hepatitis B	To assess hospitalisations, comorbidities, and costs of acute hepatitis B.	National (2001-2006)
Ascaso et al. (2024) **[50]**	*Science of The Total Environment*	Bacterial foodborne diseases (salmonellosis, campylobacteriosis, E. coli)	To analyse the effect of heatwaves and temperature on emergency admissions due to bacterial foodborne diseases.	Madrid (2013-2018)
Asensio y Monge (2012) **[51]**	*Enfermedades Infecciosas y Microbiología Clínica*	Clostridium difficile infection (CDI)	To review epidemiological data and trends in CDI in Spain.	National (1982-2011)
Ayala-Ramírez et al. (2014) **[52]**	*European Journal of Clinical Microbiology and Infectious Diseases*	Sepsis (bacterial, severe sepsis)	To analyse trends and mortality factors in sepsis hospitalisations.	Madrid (2003-2011)
Barrachina Martínez et al. (2018) **[53]**	*Revista Espanola de Salud Publica*	Chronic hepatitis C	To estimate hospitalisation costs associated with chronic hepatitis C (HCC).	Valencia (2013)
Bayas et al. (2011) **[54]**	*Vacunas*	Herpes zoster	To assess hospitalisation rates and costs of herpes zoster and postherpetic neuralgia.	Catalonia (1998-2003.)
Belhassen-García et al. (2017) **[55]**	*PLoS ONE*	Strongyloidiasis	To analyse hospitalisation burden due to strongyloidiasis.	National (1998-2014.)
García-San Miguel et al. (2014) **[56]**	*Enfermedades Infecciosas y Microbiología Clínica*	Antibiotic use and antimicrobial stewardship	To analyse adherence to antibiotic prescription recommendations.	National (2004-2005)
López-Herrero et al. (2023) **[57]**	*European Journal of Public Health*	Bacterial co-infection and SARS-CoV-2	Identify frequency and risk factors for bacterial co-infections among hospitalized COVID-19 cases	National (2020)

Rodrigo Val et al. (2007) **[58]**	*Gaceta Sanitaria*	*Bacterial meningitis (non-meningococcal)*	*Describe incidence and evaluate surveillance*	*Zaragoza (1999-2004)*
Calderón et al. (2004) **[59]**	*Clinical Microbiology and Infection*	*Pneumocystis carinii* pneumonia	To describe incidence and epidemiological features of Pneumocystis carinii pneumonia.	Andalusia (1998-1999)
Calle et al. (2006) **[60]**	*Revista de Calidad Asistencial*	Ambulatory care sensitive conditions	To identify potentially avoidable hospitalisations for ACSCs using CMBD.	Murcia (2002-2003)
Campins et al. (2013) **[61]**	*Enfermedades Infecciosas y Microbiología Clínica*	Bordetella pertussis	To review epidemiological trends and control strategies for pertussis in Spain	National (1995-2005)
Casanova Matutano et al. (1998) **[62]**	*Gaceta Sanitaria*	Ambulatory care sensitive conditions	To analyse avoidable pediatric hospitalisations and relation to primary care	Valencia & Catalonia (1993-1994)
Comes Castellano et al. (2004) **[63]**	*Revista Espanola de Salud Publica*	Pneumococcal infection	To estimate incidence and evolution of pneumococcal pneumonias	Valencia (1995-2001)
Comes Castellano et al. (2005) **[64]**	*Anales de Medicina Interna*	Pneumonia	To analyse pneumonia incidence and trends in Valencia	Valencia (1995-2001)
Condes et al. (2010) **[65]**	*Enfermedades Infecciosas y Microbiología Clínica*	HIV infection	To describe epidemiological evolution of HIV infection over 25 years	Madrid (1983-2008)
Crespo y Domínguez (2013) **[66]**	*Vacunas*	Bordetella pertussis	Analyse pertussis epidemiology and vaccination coverage.	National (2000-2011)
Culqui et al. (2015) **[67]**	*Enfermedades Infecciosas y Microbiologia Clinica*	Tuberculosis	Describe TB hospitalisation trends and demographics using CMBD.	National (1999-2009)
De Miguel-Díez et al. (2016) **[68]**	*PLoS ONE*	Chronic obstructive pulmonary disease (COPD) and HIV infection	Estimate incidence/mortality of COPD in HIV patients	National (1997-2012)
Arias-Fernández et al. (2020) **[69]**	*BMC Infectious Diseases*	Community-acquired pneumonia (CAP)	To analyse incidence, mortality, and lethality of CAP with CVD comorbidity.	National (1997-2015)
Cabré et al. (2006) **[70]**	*Medicina Clínica*	Community-acquired pneumonia (CAP)	To identify prognostic factors of community-acquired pneumonia (CAP) in elderly hospitalized patients.	Catalonia (1996)
De Miguel-Díez et al. (2020) **[71]**	*European Journal of Internal Medicine*	Pneumonia	Compare incidence and outcomes of pneumonia in COPD vs. non-COPD	National (2016-2017)
Del Carmen Álvarez-Castillo et al. (2011) **[72]**	*Gaceta Sanitaria*	Tuberculosis	Evaluate performance of TB control program in Madrid area.	Madrid (1999-2004)
Díez Domingo et al. (2006) **[73]**	*Anales de Pediatría*	Respiratory syncytial virus (RSV)	Assess incidence and costs of bronchiolitis hospitalisations.	Valencia (2001-2002)
Díez-Domingo et al. (2011) **[74]**	*Vaccine*	Streptococcus pneumoniae infection	Evaluate cost-effectiveness of universal PCV-13 vaccination.	Valencia (2009-2010)
Díez-Domingo et al. (2019) **[75]**	*Anales de Pediatría*	Rotavirus (gastroenteritis)	Review of epidemiology, burden, and vaccination experience.	National (2006-2018)
Espejo y Dols (2007) **[76]**	*Revista Espanola de Salud Publica*	Infectious diseases	To describe hospital admission incidence for infectious diseases	Valencia (1999-2003)
De Miguel-Díez et al. (2017) **[77]**	*European Journal of Internal Medicine*	Community-acquired pneumonia (CAP)	Analyse incidence and mortality trends of CAP	National (2004-2013)

De Miguel-Díez et al. (2017) **[78]**	*European Journal of Internal Medicine*	Community-acquired pneumonia (CAP)	To compare outcomes of CAP in patients with and without COPD.	National (2004-2013)
Fernández-Cano et al. (2014) **[79]**	*European Journal of Pediatrics*	Bordetella pertussis	To assess hospitalisation, mortality, and cost of pertussis	National (1997-2010)
Fernández-Cano et al. (2014) **[80]**	*Enfermedades Infecciosas y Microbiología Clínica*	Bordetella pertussis	To describe underreporting and true incidence of pertussis	National (1997-2010)
Fernández-Cano et al. (2015) **[81]**	*Vaccine*	Bordetella pertussis	To assess economic impact of new vaccination strategies	National (2009-2011)
Hinojosa Mena-Bernal et al. (2011) **[82]**	*Revista Clínica Española*	Community-acquired pneumonia (CAP)	To evaluate appropriateness of hospitalisation and treatment for CAP.	Valladolid (2006)
García Cenoz et al. (2011) **[83]**	*Anales Del Sistema Sanitario de Navarra*	Chickenpox	To evaluate impact of universal varicella vaccination.	Navarra (2006-2010)
García Comas et al. (2018) **[84]**	*Atencion Primaria*	Chickenpox	To analyse hospitalisation and incidence trends after vaccination introduction.	Madrid (2001-2015)
Jiménez-Trujillo et al. (2017) **[85]**	*Vaccine*	Community-acquired pneumonia (CAP)	To analyse temporal trends of pediatric CAP hospitalisations.	National (2001-2014)
García-Carretero et al. (2023) **[86]**	*BMC Infectious Diseases*	SARS-COV-2	To analyse hospitalisation burden and epidemiological profile of COVID-19 in Spain.	National (2020-2021)
López-de-Andrés et al. (2017) **[87]**	*BMJ Open*	Community-acquired pneumonia (CAP)	Assess trends, outcomes, and factors associated with pneumonia hospitalisations in diabetics vs non-diabetics	National (2004-2013)
García-García et al. (2010) **[88]**	*Sexual and Reproductive Healthcare*	Syphilis infection	To describe hospitalisations for syphilis in major Spanish cities.	National (1997-2006)
García-Magallón et al. (2015) **[89]**	*Revista Espanola de Salud Publica*	*Rickettsia conorii* (Mediterranean spotted fever)	To describe epidemiology and spatial distribution of MSF in Spain.	National (2009-2012)
Ochoa Gondar et al. (2013) **[90]**	*Atención Primaria*	Community-acquired pneumonia (CAP)	Validate modified CRB-75 scale for elderly CAP	Tarragona (2008-2010)
Romero Pizarro et al. (2009) **[91]**	*Revista Clínica Española*	Community-acquired pneumonia (CAP)	*Evaluate time to antibiotic administration in emergency department*	*Madrid (2007)*
Garrido-Estepa et al. (2022) **[92]**	*Gastroenterología y Hepatología (English Edition)*	Hepatitis C and co-infections (HIV, HBV, bacterial)	To describe comorbidities and co-infections in HCV-related hospitalisations.	National (2012-2019)
Gasch-Illescas et al. (2023) **[93]**	*Scientific Reports*	SARS-COV-2	To assess how COVID-19 affected hospital admissions for other conditions.	Andalusia (2017-2020)
Gea-Izquierdo et al. (2023) **[94]**	*Microorganisms*	Legionellosis	To analyse epidemiology and burden of Legionella-associated hospitalisations.	National (2002-2021)
Gea-Izquierdo et al. (2024) **[95]**	*Antibiotics*	Pneumococcal infection	To analyse pneumococcal infections in asplenic patients.	National (1997-2021)
Gea-Izquierdo et al. (2024) **[96]**	*BMC Infectious Diseases*	Pneumococcal infection	To evaluate comorbidity index performance in predicting mortality.	National (1997-2021)
Giavedoni et al. (2015) **[97]**	*Actas Dermo-Sifiliográficas*	Cutaneous leishmaniasis	To describe clinical and epidemiological characteristics of cutaneous leishmaniasis.	Barcelona (1992-2012.)

Gil et al. (2002) **[98]**	*Vacunas*	Chickenpox	To describe the hospitalisation burden of varicella before vaccine introduction.	Madrid (1995-1999)
Gil et al. (2004) **[99]**	*Vacunas*	Hepatitis A virus infection	To estimate incidence and trends of hepatitis A hospitalisations.	National (1995-1998)
Gil et al. (2005) **[100]**	*Vacunas*	Rotavirus infection	To estimate hospitalisation burden of rotavirus gastroenteritis in children.	Catalonia (1999-2000)
Gil et al. (2007) **[101]**	*Human Vaccines*	Human papillomavirus-related cancer	To estimate the burden of cervical cancer admissions in Spain.	National (1999-2002)
Gil-Prieto et al. (2011) **[102]**	*Vaccine*	Community-acquired pneumonia (pneumococcal)	To estimate burden and hospitalisation trends	National (2003-2007)
Gil de Miguel et al. (2006) **[103]**	*Anales de Pediatria*	Rotavirus (gastroenteritis)	To estimate rotavirus-related hospitalisation burden.	Madrid (1999-2000)
Gil de Miguel et al. (2022) **[104]**	*PharmacoEconomics - Open*	Pneumonia, herpes zoster, influenza, pertussis	To estimate hospitalisation costs of selected vaccine-preventable diseases.	National (2015)
Gil Prieto et al. (2006) **[105]**	*Vacunas*	Pneumonia and influenza	To quantify hospitalisation burden in middle-aged adults.	Madrid (1999-2002)
Gil Prieto et al. (2009) **[106]**	*Journal of Infection*	Salmonella infection	To analyse national trends and burden of Salmonella hospitalisations.	National (1997-2006)
Gil Prieto et al. (2009) **[107]**	*Vaccine*	Pneumococcal infection	To describe trends in pediatric pneumococcal meningitis hospitalisations.	National (1998-2006)
Gil Prieto et al. (2010) **[108]**	*Medicina Clinica*	Meningococcal infection	To analyse meningococcal hospitalisation trends in Madrid.	Madrid (1997-2005)
Gil et al. (2009) **[109]**	*Vacunas*	Bordetella pertussis	To assess hospitalisation trends for pertussis in infants.	National (1999-2005)
Gil-Prieto et al. (2009) **[110]**	*Human Vaccines*	Rotavirus infection	To analyse nosocomial rotavirus infections in Spain	National (1998-2007)
Gil-Prieto et al. (2011) **[111]**	*Vaccine*	Meningococcal infection	To evaluate hospitalisation trends and burden	National (1997-2008)
Gil-Prieto et al. (2013) **[112]**	*Vaccine*	Rotavirus infection	To assess impact of non-routine vaccination on hospitalisation rates	National (2005-2009)
Gil-Prieto et al. (2014) **[113]**	*Special Section: Vaccination Ethics*	Chickenpox	To analyse vaccination coverage and hospitalisation trends	National (2009-2010)
Gil-Prieto et al. (2014) **[114]**	*Vaccine*	Chickenpox / herpes zoster	To compare hospitalisation rates by vaccination strategy	National (2005-2010)
Gil-Prieto et al. (2019) **[94]**	*Vaccine*	Bordetella pertussis	To estimate hospitalisation burden and evaluate vaccine impact	National (1997-2017)
Gili-Miner et al. (2015) **[116]**	*Archivos de Bronconeumología (English Edition)*	Pneumococcal infection	To evaluate the effect of alcohol use disorder on outcomes of community-acquired pneumonia.	National (2008-2010)
Gili-Ortiz et al. (2015) **[117]**	*Actas Urológicas Españolas (English Edition)*	Surgical site infection	To analyse mortality, prolonged stay, and excess hospital costs due to SSIs after cystectomy.	National (2008-2010)
Giménez Duran et al. (2011) **[118]**	*Gaceta Sanitaria*	Tuberculosis	To assess underreporting of TB cases in Baleares.	Balearic islands (2005-2007)
González et al. (2003) **[119]**	*Gaceta Sanitaria*	*Invasive pneumococcal disease (IPD)*	To describe incidence trends and implications for childhood vaccination schedule.	Cantabria (1995-2001)

González et al. (2016) **[120]**	*Enfermedades Infecciosas y Microbiologia Clinica*	Pneumococcal disease	To determine hospitalisation incidence of pneumococcal disease in children with risk factors.	Catalonia (2005-2012)
González-García et al. (2021) **[121]**	*Mycoses*	Aspergillosis	To analyse trends and risk factors in aspergillosis hospitalisations.	National (1997-2017)
Guerrero-Espejo et al. (2017) **[122]**	*Acta Otorrinolaringologica (English Edition)*	Malignant external otitis	To assess incidence, mortality, and comorbidities of malignant otitis externa.	National (2008-2013)
Guerrero-Espejo y Bernad-Anso (2020) **[123]**	*Enfermedades Infecciosas y Microbiologia Clinica (English Ed)*	Fascioliasis	To describe incidence and geographic distribution of fascioliasis in Spain.	National (1997-2014)
Guillén et al. (2009) **[124]**	*Epidemiology and Infection*	Chickenpox	To analyse national burden of pediatric varicella hospitalisations.	National (1999-2005)
Guillén et al. (2010) **[125]**	*Human Vaccines*	Chickenpox	To assess hospitalisation rates in adults with varicella.	National (2001-2007)
Guillén Ortega et al. (2006) **[126]**	*Vacunas*	Varicella-zoster virus infection	To estimate hospital burden of varicella and zoster in Madrid.	Madrid (2001-2003)
Haeberer et al. (2024) **[127]**	*Vaccine*	Respiratory syncytial virus (RSV)	To estimate the economic burden of pediatric RSV hospitalisations.	National (2016-2019)
Heppe-Montero et al. (2022) **[128]**	*International Journal of Environmental Research and Public Health*	RSV and influenza infections	To assess hospitalisation and mortality burden of RSV and influenza in adults.	National (2012-2020)
Heppe-Montero et al. (2022) **[129]**	*BMC Infectious Diseases*	RSV (lower respiratory tract infections)	To quantify RSV-related pediatric hospitalisations and fatality rates.	National (2012-2018)
De Juanes et al. (2011) **[130]**	*Vacunas*	Herpes zoster / postherpetic neuralgia	Analyse frequency, costs, and complications of HZ hospitalisations	Madrid (1998-2003)
Inigo et al. (2006) **[131]**	*Medicina Intensiva*	Severe sepsis	To estimate incidence, mortality, and cost of severe sepsis hospitalisations.	Madrid (2021)
Izquierdo Carreño et al. (2003) **[132]**	*Revista Espanola de Salud Publica*	Meningococcal infection	To evaluate the surveillance sensitivity for meningococcal disease.	Tenerife (1999-2001)
Javier Luquero et al. (2009) **[133]**	*GACETA SANITARIA*	Rotavirus (gastroenteritis)	To describe hospitalisation patterns during a rotavirus outbreak.	Valladolid (2000-2005)
Jensen et al. (2012) **[134]**	*Pediatric Infectious Disease Journal*	Mycobacterial infections in HIV	To assess trends in mycobacterial disease in HIV-infected children post-HAART.	National (1997-2008)
Jiménez Caballero y Serviá Candela (2012) **[135]**	*Neurología Argentina*	Streptococcus pneumoniae	To describe epidemiological and clinical features of pneumococcal meningitis.	Toledo (2000-2008)
Jiménez Caballero et al. (2011) **[136]**	*Neurología*	Viral meningitis	To describe epidemiological and clinical differences between pediatric and adult viral meningitis cases.	Toledo (2000-2008)
Jiménez De Ory et al. (2019) **[137]**	*PLoS ONE*	Perinatal HIV infection	To assess trends and risk factors for perinatal HIV transmission in Spain.	National (1997-2015)
Jiménez-Puente et al. (2002) **[138]**	*Medicina Clinica*	Readmissions	To determine avoidable hospital readmissions.	Málaga (1997)
García-Doval et al. (2010) **[139]**	*Annals of the Rheumatic Diseases*	Herpes zoster / varicella	To assess hospitalisation risk for viral infections in patients treated with TNF antagonists.	National (2003-2006)
Muñoz-Quiles et al. (2018) **[140]**	*Journal of Infection*	Herpes zoster and postherpetic neuralgia	Describe epidemiology and burden	Valencia (2009-2014)

Latasa et al. (2018) **[141]**	*Human Vaccines and Immunotherapeutics*	Chickenpox	Evaluate vaccine impact on varicella incidence and hospitalisations.	Madrid (2001-2015)
Lema Devesa MC et al. (2002) **[142]**	*Vacunas*	Pneumococcal infection	Assess cost-benefit of discharge immunization for pneumococcal disease.	Galicia (1997-2001)
León Rubio y Espejo (2023) **[143]**	*Reumatología Clínica (English Edition)*	Osteoarticular tuberculosis	Analyse trends in osteoarticular TB hospitalisations in Spain.	National (1997-2018)
Librero y Peiró (1998) **[144]**	*Gaceta Sanitaria / SESPAS*	Hospital morbidity and mortality	Examine biases in hospital data on comorbidity and mortality.	Valencia (1993-1994)
Esteban-Vasallo et al. (2015) **[145]**	*Journal of Infection*	Herpes zoster infection	To describe temporal trends and risk profiles	Madrid (2003-2013)
López del Pino y Guerrero Espejo (2019) **[146]**	*Medicina Clínica (English Edition)*	Osteomyelitis	Estimate national incidence and mortality of osteomyelitis	National (1997-2014)
López Pérez et al. (2015) **[147]**	*Revista de Calidad Asistencial*	Pneumonia	Describe patient characteristics and factors associated with readmission in a specific DRG (pneumonia)	Madrid (2010)
López-Bernús et al. (2016) **[148]**	*Enfermedades Infecciosas y Microbiología Clínica*	Echinococcus granulosus	Describe hospital burden and trends of hydatidosis in Extremadura	Extremadura (2003-2012)
López-De-Andrés et al. (2008) **[149]**	*BMC Public Health*	Rotavirus (gastroenteritis)	Estimate national hospital burden of rotavirus infection before vaccine introduction	National (2001-2005)
Esteban-Vasallo et al. (2016) **[150]**	*Journal of Infection*	Herpes zoster infection	To analyse pre-vaccine herpes zoster hospital burden	Madrid (2003-2013)
López-Fernández et al. (2019) **[151]**	*Medicina Clínica (English Edition)*	Influenza	Examine correlation between influenza activity and CV outcomes	Castilla y León (2001-2015)
García-Rojas et al. (2017) **[152]**	*BMC Infectious Diseases*	Herpes zoster infection	To describe HZ hospitalisation trends in the Canary Islands.	Canary islands (2005-2014)
López-Herrero et al. (2024) **[153]**	*Scientific Reports*	Fungal infections and Sars-CoV2	Assess epidemiology of fungal infections in hospitalized COVID-19 patients	National (2020-2021)
Lopez-Lacort et al. (2016) **[154]**	*Vaccine*	Rotavirus (vaccine)	Evaluate apparent rotavirus cases post-vaccination	Valencia (2008-2012)
López-Lacort et al. (2024) **[155]**	*Journal of Infection and Public Health*	RSV bronchiolitis (viral infection)	Estimate the potential reduction in hospitalisations due to RSV using new prophylactic interventions	Valencia (2010-2016)
Luquero Alcalde et al. (2008) **[156]**	*European Journal of Pediatrics*	Rotavirus (gastroenteritis)	Quantify pediatric rotavirus hospitalisation burden before vaccine rollout	Valladolid (2000-2004)
Luquero et al. (2009) **[157]**	*Gaceta Sanitaria*	Rotavirus (gastroenteritis)	Describe hospital admissions during epidemic rotavirus period; develop predictive model	Valladolid (2006)
Marco-Martínez et al. (2015) **[158]**	*Enfermedades Infecciosas y Microbiologia Clinica*	*Clostridium difficile infection (CDI)*	Describe incidence, outcomes, and burden of CDI hospitalisations	National (2005-2010)
Martín et al. (2009) **[159]**	*Revista Española de Cardiología (English Edition)*	Bartonella henselae	Describe clinical characteristics of Bartonella endocarditis	National (1995-2006)
Gil et al. (2009) **[160]**	*BMC Infectious Diseases*	Herpes zoster infection	To assess incidence and hospitalisation trends of herpes zoster.	National (1998-2004)
Mate-Cano et al. (2020) **[161]**	*European Journal of Internal Medicine*	Hepatitis C virus infection	Describe national trends in HCV-related hospitalisations and outcomes	National (2000-2015)

Mayoral Cortés et al. (2001) **[162]**	*European Journal of Epidemiology*	Pulmonary tuberculosis and HIV coinfection	Estimate the incidence and overlap of TB and HIV	Seville (1998)
Medrano et al. (2014) **[163]**	*Critical Care*	HIV infection, chronic hepatitis C, sepsis	Assess ICU mortality among HIV patients and the role of sepsis	National (2005-2010)
Meléndez Frigola et al. (2016) **[164]**	*Revista Espanola de Salud Publica*	General subacute hospitalisations	Characterise subacute patients using CMBD-RSS	Girona (2013-2014)
Mérida et al. (2011) **[165]**	*Medicina Clínica*	HIV and renal outcomes	Evaluate ART protection against renal deterioration in HIV patients	Andalusia (2007-2008)
Molina-Salas et al. (2018) **[166]**	*Enfermería Clínica (English Edition)*	Postsplenectomy infection risk (pneumococcal, meningococcal, Hib)	Assess vaccination coverage and infection risk post-splenectomy	Murcia (1993-2012)
Monreal et al. (2020) **[167]**	*Enfermedades Infecciosas y Microbiologia Clinica (English Ed)*	HIV and stroke	Evaluate prevalence and outcomes of HIV in stroke hospitalisations	National (1997-2012)
Montes-Santiago et al. (2010) **[168]**	*Enfermedades Infecciosas y Microbiología Clínica*	Tuberculosis	Analyse hospitalisations and costs related to TB in Spain	National (1999-2006)
Morano et al. (2011) **[169]**	*GACETA SANITARIA*	Pneumococcal infection	Evaluate cost-effectiveness of pneumococcal vaccination	National (1990-2009)
Moreno et al. (2023) **[170]**	*JMIR Research Protocols*	*Urinary tract infection (UTI)*	Protocol for cohort study on diagnosis and treatment of UTIs	Catalonia (2012-2021)
Moreno Carrillo et al. (2022) **[171]**	*Rehabilitación*	Diabetic foot	Evaluate outcomes of multidisciplinary diabetic foot unit	Murcia (2010-2020)
Moreno-Iribas et al. (2005) **[172]**	*Gaceta Sanitaria*	HIV infection	Assess surveillance methods for HIV infection in Navarra	Navarra (1991-2003)
Moreno-Pérez et al. (2005) **[173]**	*Vacunas*	*Invasive pneumococcal disease (IPD)*	Describe epidemiology in children <5 years old	Málaga (1999-2001)
Moreno-Torres et al. (2023) **[174]**	*International Journal of Infectious Diseases*	SARS-CoV-2	Evaluate mortality in SOT recipients hospitalized with COVID-19	National (2020)
Morral-Puigmal et al. (2018) **[175]**	*Environment International*	Gastroenteritis (bacterial, viral, idiopathic)	Assess association between weather (temperature, rainfall) and gastroenteritis	National (1997-2013)
Mostaza et al. (2022) **[176]**	*Revista Clínica Española (English Edition)*	SARS-CoV-2	Identify predictors of mortality in elderly COVID-19 patients	Madrid (2020-2021)
Muñoz (2024) **[177]**	*Heart & Lung*	SARS-CoV-2	Assess effects of COVID-19 on non-COVID ventilation cases	National (2020-2021)
Muñoz Martínez et al. (2006) **[178]**	*Revista Clínica Española*	Legionella pneumonia	Describe outbreak and clinical management	Vigo (2000)
Muñoz-Moreno et al. (2019) **[179]**	*Journal of Clinical Medicine*	Infective endocarditis and HIV	Evaluate national trends of IE among HIV patients	National (1997-2014)
Gil-Prieto et al. (2011) **[180]**	*Vacunas*	Herpes zoster infection	To assess herpes zoster hospitalisation burden in chronic disease patients	National (1998-2004)
Muñoz-Quiles et al. (2022) **[181]**	*Vaccine*	Human papillomavirus (vaccine)	Assess effectiveness of HPV vaccine	Valencia (2009-2017)
Núñez et al. (2007) **[182]**	*Gastroenterología y Hepatología*	Liver diseases	Quantify hepatology burden in Spain and hospitals	Madrid (1999-2003)

Llácer et al. (2014) **[183]**	*Gaceta Sanitaria*	Infectious diseases in general	Discuss the impact of the economic crisis on infectious diseases in Spain	National (2005-2007, 2009-2011)
Ochoa-Gondar et al. (2023) **[184]**	*Atención Primaria*	Pneumococcal infection	Assess incidence and outcomes of pneumococcal pneumonia	Catalonia (2017-2018)
Otero et al. (2023) **[185]**	*REVISTA DE SALUD AMBIENTAL*	Campylobacter jejuni	Describe national burden of campylobacteriosis	National (2013-2019)
Otero et al. (2024) **[186]**	*Revista Espanola de Nutricion Humana y Dietetica*	Salmonellosis	Describe national epidemiology before COVID-19	National (2013-2019)
Palacios-Ceña et al. (2017) **[187]**	*European Journal of Internal Medicine*	Aspiration pneumonia	Assess national trends in incidence and mortality of aspiration pneumonia in elderly	National (2003-2013)
Palacios-Fernandez et al. (2021) **[188]**	*BMC Geriatrics*	All-cause hospitalisation	Describe hospital discharge trends in oldest-old	National (2000-2015)
Parra Fariñas et al. (2022) **[189]**	*Mycoses*	Mucormycosis	Assess national incidence and trends of mucormycosis	National (1997-2018)
Pedraz et al. (2024) **[8]**	*PLoS ONE*	Tuberculosis	Compare surveillance vs hospitalisations	National (2012-2020)
Pérez Arbej et al. (2010) **[190]**	*Actas Urológicas Españolas (English Edition)*	Surgical site infection	Evaluate SSI rate in urology	Zaragoza (2004-2007)
Pérez Rojo et al. (2015) **[191]**	*Vacunas*	Rotavirus (gastroenteritis)	Measure burden and hospitalisation rates of rotavirus in children <5 years	Salamanca (2008-2013)
Pérez Zapata et al. (2017) **[192]**	*Revista de Calidad Asistencial*	*Adverse events (surgery)*	Compare Trigger Tool vs CMBD to detect surgical adverse events	N.A. (2012)
Barba-Martin et al. (2023) **[193]**	*Revista Clínica Española (English Edition)*	Infectious diseases including SARS-CoV-2	To describe infectious disease hospitalisations before and during COVID-19 pandemic.	National (2016-2020)
Pérez-Rubio et al. (2024) **[194]**	*BMC Infectious Diseases*	Multiple vaccine-preventable infections (rotavirus, influenza, etc.)	Quantify health burden (DALYs) of 12 preventable diseases	National (2016-2019)
Pérez-Vilar et al. (2014) **[195]**	*Anales de Pediatría (English Edition)*	Rotavirus (vaccine)	Estimate underreporting of adverse events post-vaccination	Valencia (2007-2011)
Puig-Barberà et al. (2013) **[196]**	*Vaccine*	*Influenza*	*Compare effectiveness of two influenza vaccines in older adults*	*Valencia (2010-2011)*
Quirós-González et al. (2023) **[197]**	*Revista Española de Quimioterapia*	*Influenza*	*Validate RAE-CMBD data vs. epidemiological influenza registry*	*Salamanca (2017-2019)*
Ramos et al. (2015) **[198]**	*Enfermedades Infecciosas y Microbiología Clínica*	*Infectious diseases (TB, HIV, malaria, hepatitis, UTI)*	*Characterise infections leading to hospitalisation among foreign adults*	*Alicante (2000-2012)*
Ramos-Rincón et al. (2023) **[199]**	*Journal of Clinical Virology*	*Viral hepatitis and SarsCov2*	*Assess change in hepatitis-related hospitalisations during COVID-19*	*National (2019-2020)*
Redondo et al. (2015) **[200]**	*Human Vaccines and Immunotherapeutics*	*Rotavirus (gastroenteritis)*	*Assess vaccine impact on rotavirus hospitalisations*	*Castilla-La Mancha (2003-2009)*
Redondo Sánchez et al. (2023) **[201]**	*Anales de Pediatría*	*Urinary tract infection (UTI)*	*Assess hospitalisation trends for UTI in children*	*National (2000-2015)*

Redondo-Calvo et al. (2024) **[202]**	*Journal of Proteome Research*	*SARS-CoV-2*	*Analyse persistent inflammation after SARS-CoV-2*	*Ciudad Real (2021)*
Redondo-González (2015) **[203]**	*Revista Espanola de Enfermedades Digestivas*	*Rotavirus (nosocomial infection)*	*Validate CMBD vs. microbiological registry for nosocomial gastroenteritis*	*Guadalajara (2003-2009)*
Redondo-González & Tenías-Burillo (2016) **[204]**	*Epidemiology and Infection*	*Rotavirus (community-acquired)*	*Identify features associated with rotavirus hospitalisation*	*Castilla-La Mancha (2003-2009)*
Redondo-González & Tenías-Burillo (2017) **[205]**	*Epidemiology and Infection*	*Rotavirus*	*Assess effect of vaccine introduction on rotavirus burden (self-financed vaccines)*	*Castilla-La Mancha (2003-2009)*
Riu et al. (2012) **[206]**	*Enfermedades Infecciosas y Microbiología Clínica*	*Nosocomial bacteremia*	*Quantify excess hospital stay and cost of bacteremia*	*Barcelona (2005-2007)*
Riu et al. (2016) **[207]**	*Enfermedades Infecciosas y Microbiología Clínica*	*Nosocomial bacteremia*	*Compare cost estimates from 3 economic methodologies*	*Barcelona (2005-2007)*
Martín-Aspas et al. (2007) **[208]**	*Enfermedades Infecciosas y Microbiología Clínica*	Infectious meningitis	Evaluate adherence to clinical guidelines in bacterial/viral meningitis	Huelva (1987-2004)
Rodríguez Alonso et al. (2021) **[209]**	*European Journal of Clinical Microbiology and Infectious Diseases*	*Bartonella henselae*	*Describe national trends, hospitalisation burden*	*National (1997-2015)*
Rodríguez Vidigal et al. (2020) **[210]**	*Revista Clínica Española (English Edition)*	*Hemophagocytic syndrome (HPS)*	*Characterise infection-related HPS cases*	*National (2013-2017)*
Rodríguez-Alonso et al. (2020) **[211]**	*International Journal of Infectious Diseases*	*Coxiella burnetii*	*Analyse trends and regional distribution of Q fever hospitalisations*	*National (1997-2015)*
Rodríguez-Alonso et al. (2020) **[212]**	*International Journal of Infectious Diseases*	*Rickettsia typhi (Murine typhus)*	*Assess incidence and outcomes of hospitalized murine typhus*	*National (1997-2015)*
Rodríguez-Alonso et al. (2021) **[213]**	*Epidemiology and Infection*	*Brucella spp.*	*Describe epidemiology and hospitalisation trends of brucellosis in Spain*	*National (1997-2015)*
Rodríguez-Caulo et al. (2021) **[214]**	*Cirugía Cardiovascular*	*SARS-CoV-2*	*Assess the pandemic impact on cardiac surgery activity*	*National (2019-2020)*
Rodríguez-Caulo et al. (2021) **[215]**	*Cirugía Cardiovascular*	*SARS-CoV-2*	*Quantify the impact on cardiac surgery via DRG data*	*National (2019-2020)*
Rodríguez-Martín et al. (2016) **[216]**	*Revista de Calidad Asistencial*	*Clostridium difficile infection (CDI)*	*Assess adherence to clinical guidelines*	*Valladolid (2010-2012)*
Rodríguez-Vidigal et al. (2014) **[217]**	*Revista Clínica Española (English Edition)*	*Leptospirosis*	*Describe epidemiological and clinical features in hospitalised cases*	*Badajoz (1997-2013)*
Bustamante-Munguira et al. (2018) **[218]**	*Interactive Cardiovascular and Thoracic Surgery*	Infective endocarditis	To describe surgical treatment patterns and outcomes in acute infective endocarditis.	National (1997-2014)
Salas et al. (2019) **[219]**	*Vaccine*	*Rotavirus vaccination*	*Assess whether rotavirus vaccination reduces seizure hospitalisations*	*National (2002-2015)*
Salas Jarque et al. (2023) **[220]**	*Anales Del Sistema Sanitario de Navarra*	*SARS-CoV-2*	*Assess the association of corticosteroid treatment and circulating SARS-CoV-2 variants with in-hospital mortality.*	*Huesca (2020-2021)*
Salazar et al. (2013) **[221]**	*Revista Espanola de Salud Publica*	*All-cause emergency case-mix*	*Describe and classify the case-mix of emergency department attendances using the Andalusian minimum data set.*	*Andalusia (2012)*

San Roman Montero et al. (2009) **[222]**	*Journal of Infection*	*Neisseria meningitidis*	*Describe trends in hospitalisations and in-hospital mortality due to meningococcal infection in Spain.*	*National (1997-2005)*
San-Román-Montero et al. (2019) **[223]**	*BMC Infectious Diseases*	*Influenza*	*Assess how ICD-9-CM coding position/selection for influenza relates to in-hospital fatality and patient characteristics.*	*National (2009-2015)*
Sánchez-de Prada et al. (2023) **[224]**	*Environmental Research*	*SARS-COV-2*	*Evaluate associations between environmental factors and hospital outcomes among COVID-19 inpatients.*	*National (2020)*
Santos et al. (2008) **[225]**	*Vacunas*	*Bordetella pertussis*	*Estimate the burden and trends of hospitalisations attributable to pertussis in Spain.*	*National (1999-2005)*
Santos-Sancho et al. (2010) **[226]**	*Anales del Sistema Sanitario de Navarra*	*Rubella*	*Describe the epidemiology and trends of rubella-related hospitalisations in Spain.*	*National (1997-2006)*
Santos-Sancho et al. (2010) **[227]**	*Journal of Pediatric Infectious Diseases*	*Measles*	*Estimate incidence, LOS, mortality and costs of measles hospitalisations in Spain.*	*National (1997-2006)*
Santos-Sancho et al. (2011) **[228]**	*Journal of Pediatric Infectious Diseases*	*Mumps*	*Estimate incidence, LOS, mortality and costs of mumps hospitalisations.*	*National (1997-2006)*
Zulet et al. (2024) **[229]**	*Revista Española de Cardiología*	Infective endocarditis	*Describe regional incidence, characteristics, outcomes.*	*National (2016-2019)*
Sarría-Santamera et al. (2023) **[230]**	*International Journal of Environmental Research and Public Health*	*Postoperative sepsis*	*Estimate postoperative sepsis incidence in elderly surgical patients and assess HFRS predictive capacity.*	*National (2016-2018)*
Servia-Dopazo et al. (2020) **[231]**	*Gaceta Sanitaria*	Respiratory syncytial virus (RSV)	*Assess whether a 4-hospital microbiological surveillance system captures RSV seasonality for the entire region.*	*Galicia (2008 - 2017)*
SETH (2011) **[232]**	*Cirugía Española (English Edition)*	*Hepatitis C virus infection*	*Provide consensus recommendations on HCV in LT, living donor LT, graft/program quality.*	*National (2010)*
Tamayo-Velasco et al. (2024) **[233]**	*Journal of Infection and Public Health*	*Fungal infection in hematologic patients with Sars-CoV2*	*Estimate burden and outcomes of CAPA and explore role of prophylaxis in hematologic inpatients*	*National (2020-2021)*
Tejero Encinas et al. (2003) **[234]**	*Revista Espanola de Salud Publica*	*Tuberculosis*	*Evaluate specialized-care TB surveillance using two data sources.*	*Valladolid (1996-2000)*
Uriona Tuma et al. (2013) **[235]**	*Medicina Clínica*	*Bordetella pertussis*	*Describe contact tracing of pediatric pertussis cases in tertiary hospital*	*Barcelona (2005-2009)*
Val et al. (2007) **[236]**	*Gaceta Sanitaria*	*Non-meningococcal bacterial meningitis*	*Describe epidemiology and evaluate surveillance completeness.*	*Zaragoza (1999-2004)*
Valcárcel et al. (2008) **[237]**	*Enfermedades Infecciosas y Microbiología Clínica*	*Leishmaniasis*	*Describe epidemiology of leishmaniasis hospitalisations in Spain.*	*National (1999-2003)*
Varas Vicente et al. (2023) **[238]**	*Journal of Infection and Public Health*	*Pneumocystis jirovecii pneumonia*	*Compare HIV vs non-HIV pneumocystosis hospitalisations.*	*National (1997-2020)*
Ventura Cerdá et al. (2007) **[239]**	*Medicina Clínica*	*Surgical site infection prevention*	*Assess efficacy of single-dose prophylaxis.*	*Valencia (1995-2003)*
Vila-Corcoles et al. (2020) **[240]**	*Vaccine*	Pneumococcal infection	*Evaluate vaccine-effectiveness (VE) of PCV13/PPSV23 in preventing pneumococcal disease and mortality.*	*Tarragona (2015-2016)*
Vila-Córcoles et al. (2015) **[241]**	*Enfermedades Infecciosas y Microbiología Clínica*	*Invasive pneumococcal disease (IPD)*	*Estimate IPD incidence and lethality in Tarragona*	*Tarragona (2006-2009)*

Vila-Córcoles et al. (2023) **[242]**	*Vaccine*	Pneumococcal infection	*Assess adult PCV13/PPSV23 effectiveness post-PCV13 childhood rollout*	*Catalonia (2017-2018)*
Visser (1999) **[243]**	*Acta Paediatrica*	Rotavirus (gastroenteritis)	*Estimate rotavirus-attributable admissions*	*National (1994)*
Yébenes et al. (2017) **[244]**	*Annals of Intensive Care*	*Sepsis*	*Estimate incidence and in-hospital mortality trends for sepsis*	*Catalonia (2008-2012)*
