# Supplementary material for: Three Decades of Use of the Minimum Basic Data Set in Infectious Disease Research in Spain: A Scoping Review with an Evidence-Mapping Approach
Source: Trop Med Infect Dis. 2026 Feb 20;11(2):61. doi: 10.3390/tropicalmed11020061 (PMC12945255; doi:10.3390/tropicalmed11020061)
Supplement: Supplementary file 1 [file tropicalmed-11-00061-s001.zip › Table S2. Infections as a secondary outcome or result.pdf]

Table S2. Studies included and characteristics. Infections as a secondary outcome or result

Study ID		Main Findings		
**Author (year) [Ref.]**	**Journal**	**Condition studied**	**Study Objective**	**Study región (and period)**
***Abizanda et al. (2004)* [245]**	Revista de Calidad Asistencial	Critical care	Design and test a combined indicator of effectiveness and efficiency in ICU patients.	Castellón (1998-2000)
***Ahijado-Porres et al. (2011)* [246]**	*Enfermeria Clinica*	Pregnancy-related complications	To analyse hospitalisations due to pregnancy complications in Spain from 1997-2007.	National (1997-2007)
***Alameda y Suárez (2009)* [247]**	*European Journal of Internal Medicine*	Heart failure	To compare outcomes between outlier vs non-outlier hospitalisations in heart failure patients.	Madrid (2006)
***Alfonso et al. (2022)* [248]**	*Revista Española de Cardiología (English Edition)*	Cardiovascular disease	To assess mortality and 30-day readmission in AMI-SCAD vs AMI-non-SCAD using CMBD.	National (2016-2019)
***Allepuz et al. (2013)* [249]**	*Revista Española de Cirugía Ortopédica y Traumatología*	Orthopedic prosthesis	To describe the structure and operation of the Catalan Arthroplasty Registry (RACat) as a post-marketing surveillance system.	Catalonia (2005-2011)
***Allué et al. (2014)* [250]**	*Gaceta Sanitaria*	Adverse events	Estimate the incidence and cost of adverse events in Spanish hospitals using CMBD data.	National (2008-2010)
***Álvarez Hernández et al. (2010)* [251]**	*Actividad Dietética*	Malnutrition	To discuss the importance of accurate hospital malnutrition coding for clinical management and cost analysis.	National (n.a.)
***Álvarez-Bartolomé et al. (2024)* [252]**	*REC: CardioClinics*	Valvular heart disease	To describe national burden, outcomes, and costs of valvular heart procedures in Spain.	National (2016-2019)
***Álvarez-Ibáñez y Guerra-García (2012)* [253]**	*Atención Primaria*	Upper gastrointestinal bleeding	To analyse temporal relationship between anti-ulcer medication use and incidence of upper gastrointestinal bleeding.	National (2000-2008)
***Aparicio et al. (2020)* [254]**	*Revista Clínica Española (English Edition)*	Down syndrome and respiratory infections	To describe characteristics of hospitalized adults with Down syndrome in internal medicine services.	National (2005-2014)
***Aparicio et al. (2023)* [255]**	*Journal of Applied Research in Intellectual Disabilities*	Down syndrome	To analyse causes and trends of hospital admissions in adults with Down syndrome.	National (1997-2014)
***Arias-de la Torre et al. (2017)* [256]**	*Revista Española de Cirugía Ortopédica y Traumatología (English Edition)*	Arthroplasty surveillance	To evaluate variability and survival of hip and knee prostheses.	Catalonia (2005-2014)
***Zapatero Gaviria et al. (2010)* [257]**	*Revista Clínica Española*	Adverse drug events (ADE)	Identify ADEs coded in CMBD.	National (2005-2007)
***Pérez-Blanco et al. (2004)* [258]**	*Revista de Calidad Asistencial*	Adverse drug events (ADE)	Describe adverse drug reactions captured by CMBD	Madrid (2003)
Saralegui Ansorena et	*Cirugía Española (English Edition)*	Anal squamous-cell carcinoma	Compare characteristics and oncologic outcomes between immunocompromised vs	National (2012-2017)

***al. (2022)* [259]**			immunocompetent patients with anal SCC.	
***Barrientos Vega (2003)* [260]**	*Medicina Intensiva*	Intensive care management (DRGs)	To assess the implementation and use of Diagnosis-Related Groups (GRD/CMBD) in an ICU unit for clinical and economic evaluation.	Toledo (2001)
***Barrionuevo-Sánchez et al. (2023)* [261]**	*Hellenic Journal of Cardiology*	Cardiogenic shock	To explore how etiology and hospital features affect management and prognosis.	National (2016-2019.)
***Bayas et al. (2008)* [262]**	*Vacunas*	Human papillomavirus-related cancer	To estimate hospitalisation frequency and costs of cervical cancer and carcinoma in situ.	Catalonia (1999-2002.)
***Baztán Cortés et al. (2016)* [263]**	*Revista Española de Geriatría y Gerontología*	Pneumonia, urinary infection, etc.	To analyse prognostic value of main admission diagnosis in elderly patients.	Madrid (2009.)
***Ben Cheikh et al. (2011)* [264]**	*Gaceta Sanitaria*	General morbidity	To describe hospitalisation rates among foreign-born populations.	Aragón (2004-2007.)
***Benavides et al. (2011)* [265]**	*Atención Primaria*	Occupational diseases	To identify diseases potentially work-related using national data.	Catalonia (2008)
***Blanco et al. (2023)* [266]**	*Revista Española de Cirugía Ortopédica y Traumatología*	Orthopedic trauma	To evaluate the impact of hip hemiarthroplasty dislocation on postoperative mortality after hip fracture.	Castilla y León (2010-2015)
***Brito-Zerón et al. (2023)* [267]**	*eClinicalMedicine*	Autoimmune disease	To identify mortality predictors in primary Sjögren syndrome using multicenter registry data.	International (multicenter). (Until 2014)
***Cabrera Torres et al. (2019)* [268]**	*Gaceta Sanitaria*	Hospital management	To study ectopic admissions (outlier patients) and their relation to complications and length of stay.	Salamanca (2014-2015)
***Caminal y Silvestre (2003)* [269]**	*Revista de Calidad Asistencial*	Hospital management	To describe relationship between primary care and hospital activity.	Catalonia (1998-1999)
***Campillo-Soto et al. (2005)* [270]**	*Archivos de Bronconeumología*	Spontaneous pneumomediastinum	To describe characteristics of patients with spontaneous pneumomediastinum.	Murcia (1990-2003.)
***Canora-Lebrato et al. (2012)* [271]**	*Revista Clínica Española*	Systemic lupus erythematosus (SLE)	To analyse hospital discharges for SLE in Spain using CMBD data	National (2005-2008)
***De Miguel-Díez et al. (2013)* [272]**	*Respiratory Medicine*	Chronic obstructive pulmonary disease (COPD)	Evaluate incidence, comorbidities, mortality trends	National (2006-2010)
***Casanovas et al. (2024)* [273]**	*Psychiatry Research*	Cancer	To analyse cancer characteristics and outcomes in patients with schizophrenia	Catalonia (1997-2021)
***Company-Sancho et al. (2017)* [274]**	*Enfermería Clínica*	Nursing diagnoses / chronic care	To analyse association between nursing diagnoses and healthcare costs	Canary islands (2012-2013)
***Conesa et al. (2003)* [275]**	*Gaceta Sanitaria*	Emergency care classification	To analyse and classify emergency hospitalisations by Ambulatory Patient Groups (APG).	Barcelona (1999-2000)
***Córdoba et al. (2013)* [276]**	*Actas Dermo-Sifiliográficas (English Edition)*	Post-surgical infection	To evaluate surgical activity, outcomes, and complications in dermatology.	Madrid (2005-2010)
***Cortázar García et al. (2016)* [277]**	*Radiología*	Acute cholecystitis	Evaluate percutaneous cholecystostomy outcomes in	Málaga (2011-2013)

			acute cholecystitis high-risk patients.	
***Cortiñas Sáenz et al. (2007)* [278]**	*Cirugía Española*	Minor postoperative infections	Quantify readmissions due to complications in ambulatory surgery.	Albacete (1998-2003)
***Cortiñas Saenz et al. (2012)* [279]**	*Actas Dermo-Sifiliográficas (English Edition)*	Minor postoperative infections	Assess results and complications in outpatient dermatologic surgery.	Almeria (2004-2007)
***Cots et al. (2013)* [280]**	*Archivos de Bronconeumología*	Chronic respiratory infections	Assess efficiency and resource allocation of respiratory day hospital.	Barcelona (2007-2008)
***Cruz-Rojo et al. (2011)* [281]**	*Gaceta Sanitaria*	Hospital utilization patterns	Describe hospitalisation patterns in Algeciras and compare with other regions.	Cádiz (2001-2005)
***Daigre et al. (2007)* [282]**	*Mujer y Salud Mental*	Infections secondary to substance use (HIV, hepatitis)	Analyse comorbidities of mental and substance-related disorders in women.	Catalonia (2003)
***De Juanes et al. (2010)* [283]**	*Progresos de Obstetricia y Ginecologia*	Cervical cancer	Assess frequency, characteristics, and costs of cervical cancer hospitalisations	Madrid (1999-2002)
***Fernández-García et al. (2022)* [284]**	*Revista Clínica Española (English Edition)*	Chronic obstructive pulmonary disease (COPD)	To analyse evolution of hospitalisations with and due to COPD	Galicia (1996-2018)
***De la Rosa Carrillo y Prados Sánchez (2020)* [285]**	*Open Respiratory Archives*	Bronchiectasis	Review epidemiological trends of bronchiectasis in Spain	National (2002-2019)
***García-Aymerich et al. (2009)* [286]**	*Archivos de Bronconeumología*	Chronic obstructive pulmonary disease (COPD)	To describe design and methodology of the PAC-COPD cohort.	National (2004-2006)
***De Miguel-Díez et al. (2014)* [287]**	*Respiratory Medicine*	Asthma exacerbations	Assess incidence, cost, and mortality	National (2002-2010)
***De Miguel-Díez et al. (2016)* [288]**	*European Journal of Internal Medicine*	Pulmonary embolism	To examine environmental and meteorological risk factors for pulmonary embolism.	National (2001-2013)
***De Miguel-Yanes et al. (2016)* [289]**	*Hepatobiliary & Pancreatic Diseases International*	Cholecystitis	To assess differences in outcomes between open and laparoscopic cholecystectomies by diabetes status.	National (2003-2013)
***De-Miguel-Díez et al. (2018)* [290]**	*International Journal of Cardiology*	Pulmonary hypertension	To analyse national hospitalisation trends and mortality.	National (2001-2014)
***Díez et al. (1996)* [291]**	*Medicina Clinica*	General medicine	To analyse internal medicine hospital activity and efficiency using GRDs.	National (1992)
***Doval Alcalde et al. (2023)* [292]**	*Journal of Healthcare Quality Research*	Pediatric acute admissions	To analyse pediatric "zero-stay" hospitalisations over 25 years.	N.A. (1993-2017)
***Escobar et al. (2011)* [293]**	*Revista Clínica Española*	Day hospital management	To assess avoided admissions via day hospital	Cádiz (2010-2011)
***Fernandez-Alonso et al. (2024)* [294]**	*Human Vaccines and Immunotherapeutics*	Anus and penis cancer	To quantify hospitalisations and costs related to HPV-associated cancers	National (2016-2020)
***Freixinet Gilart et al. (2016)* [295]**	*Archivos de Bronconeumología (English Edition)*	Thoracic surgery indicator	To assess outcomes and quality indicators in thoracic surgery across Spanish hospitals.	National (2002-2003)
Galán González-	*Archivos de Bronconeumología*	Respiratory diseases (includes infections)	To evaluate efficiency in pulmonary units by hospital size.	Andalusia (1994-1995)

***Sema et al. (1999)* [296]**				
***Galán et al. (2018)* [297]**	*Revista Española de Cardiología (English Edition)*	Cardiovascular disease	To evaluate impact of smoking bans on cardiovascular admissions.	National (2003-2012)
***Galindo Mateu et al. (2010)* [298]**	*Progresos de Obstetricia y Ginecología*	Obstetric complications	To analyse near-miss obstetric cases and their causes.	Valencia (1971-2007)
***Gallego et al. (2023)* [299]**	*International Journal of Environmental Research and Public Health*	Familial Mediterranean Fever (FMF)	To analyse spatial and temporal patterns of FMF hospitalisations in Spain.	National (2008-2015)
***García Rodríguez et al. (2022)* [300]**	*Endocrinología, Diabetes y Nutrición*	Malnutrition/dysphagia	To assess effects of dysphagia and malnutrition on survival in hospitalized patients.	National (2018-2019)
***García-Altés et al. (2009)* [301]**	*Gaceta Sanitaria*	Health system performance	To assess performance of Catalan healthcare services.	Catalonia (2005)
***García-Cornejo y Pérez-Méndez (2018)* [302]**	*Gaceta Sanitaria*	Economic analysis	To analyse relationship between cost systems and hospital expenditure.	National (2010-2015)
***García-Cubillo et al. (2006)* [303]**	*Revista de Calidad Asistencial*	Integrated care processes	Describe and implement an evaluation model for Integrated Care Processes (PAI) using CMBD data and quality indicators.	Andalusia (2004)
***García-Garrido et al. (2014)* [304]**	*Gaceta Sanitaria*	Human papillomavirus-related cancer	To estimate coverage and costs of opportunistic cervical cancer screening.	Cantabria (2006-2011)
***García-Tirado et al. (2019)* [305]**	*Cirugía Española*	Pulmonary resection (admisions)	To review evidence on unplanned readmissions after pulmonary resection.	National (until 2017)
***Gil-Bona et al. (2010)* [306]**	*Cirugía Española (English Edition)*	Surgical mortality risk assessment	To analyse the predictive capacity of the Charlson Comorbidity Index and Surgical Risk Scale for surgical mortality	Catalonia (2004-2007)
***Lara-Rojas et al. (2019)* [307]**	*European Journal of Internal Medicine*	Diabetes and comorbid infections (indirect)	Assess national trends in diabetes hospitalisations and complications.	National (1997-2010)
***Carrera-Lasfuentes et al. (2015)* [308]**	*Gaceta Sanitaria*	Diabetes mellitus	To determine comorbidity predictors of mortality and healthcare use in diabetic patients	Zaragoza (2006-2010)
***Gili-Miner et al. (2018)* [309]**	*Neurología (English Edition)*	Alcohol-related disorders; Multiple sclerosis	To assess the impact of alcohol use disorder on hospital outcomes among patients with multiple sclerosis.	National (2008-2010)
***Gisbert et al. (2007)* [310]**	*Gaceta Sanitaria*	Health expenditure	To distribute healthcare budget by ICD-9-CM diagnostic categories.	Catalonia (2005)
***Gómez-Garrido y González-Viejo (2010)* [311]**	*Rehabilitación*	Spinal cord injury	To classify acute SCI cases using DRGs.	Barcelona (1997-2006)
***Gómez-Méndez et al. (2018)* [312]**	*Medicina Clínica (English Edition)*	Alcohol withdrawal syndrome	To assess national incidence and trends of AWS hospitalisations.	National (1999-2010)

***Gómez-Rosado et al. (2019)* [313]**	*Cirugía Española (English Edition)*	Post-surgical complications (non-infectious)	To assess complications and costs among outlying surgical patients.	Andalusia (2015)
***González-Guerrero et al. (2005)* [314]**	*Revista Española de Geriatría y Gerontología*	Hospital efficiency	To assess bed management efficiency in a geriatric unit.	Caceres (2002)
***González-Guerrero et al. (2008)* [315]**	*Revista Española de Geriatría y Gerontología*	Functional dependency	To analyse case-mix of geriatric patients according to dependency.	Caceres (2002-2003)
***González-Montalvo et al. (2019)* [316]**	*Revista Española de Geriatría y Gerontología*	Hospital efficiency	To compare acute geriatric units to other departments using DRG-adjusted data.	Madrid (2011-2015)
***González-Samartino et al. (2018)* [317]**	*Revista Da Escola de Enfermagem*	Adverse events (general)	To evaluate data quality and completeness of event recording.	Catalonia (2015)
***Guerrero Espejo et al. (2023)* [318]**	*Revista Clínica Española (English Edition)*	Wiskott-Aldrich syndrome (rare immunodeficiency)	To determine national incidence and survival of WAS in Spain.	National (1997-2017)
***Guijarro et al. (2014)* [319]**	*European Journal of Internal Medicine*	Thromboembolism (non-infectious)	To analyse VTE and bleeding among medical inpatients.	National (2005-2006)
***Guijarro et al. (2016)* [320]**	*European Journal of Internal Medicine*	Cardiovascular disease	To compare outcomes of PE, ACS, and stroke.	National (2001-2010)
***Hernando Arizaleta et al. (2009)* [321]**	*Gaceta Sanitaria*	General hospitalisations	To evaluate healthcare use and economic impact of immigration on hospital activity.	Murcia (2004-2005)
***Hernando Ortiz et al. (2012)* [322]**	*Gaceta Sanitaria*	Healthcare management	To assess performance and cost-effectiveness of a day-hospital model.	Valladolid (2009)
***Jiménez Puente et al. (2010)* [323]**	*Revista Clínica Española*	General hospital case mix	To describe clinical information systems for patient classification.	National (n.a.)
***Jiménez et al. (2017)* [324]**	*Endocrinología, Diabetes y Nutrición*	Diabetic foot	To evaluate amputation trends after multidisciplinary diabetic foot unit implementation.	Madrid (2001-2014)
***Jurado-Campos et al. (2012)* [325]**	*Atención Primaria*	Diabetic foot	Identify improvement areas in diabetic foot care across primary and secondary care; describe hospital models and their relation to amputations.	Catalonia (2008)
***(307)Lázaro et al. (2012)* [326]**	*Revista Española de Geriatría y Gerontología*	Geriatric hospitalisations	Describe case mix and outcomes of ≥90-year-old patients.	National (2005-2008)
***Lecumberri et al. (2011)* [327]**	*Journal of Thrombosis and Haemostasis*	Venous thromboembolism (hospital-acquired)	Evaluate cost-effectiveness of an alert system to reduce VTE events.	Navarra (2006-2009)
***Llorente et al. (2017)* [328]**	*Actas Urológicas Españolas*	Postoperative outcomes after radical cystectomy	Assess variability in complications and mortality following radical cystectomy	National (2011-2013)
***López et al. (2017)* [329]**	*Human Vaccines and Immunotherapeutics*	Human papillomavirus-related cancer	Quantify hospitalisation burden and outcomes for anal and penile neoplasia	National (2009-2013)
***López et al. (2018)* [330]**	*Papillomavirus Research*	Human papillomavirus-related cancer	Quantify hospitalisation burden for vulvar and vaginal neoplasia	National (2009-2013)

***López-Cepero et al. (2005)* [331]**	*Gastroenterología y Hepatología*	Upper gastrointestinal bleeding	Assess association between climate factors and GI bleeding admissions	Jerez de la Frontera (1998-2001)
***López-de-Andrés et al. (2011)* [332]**	*Diabetes Research and Clinical Practice*	Gestational diabetes	Describe national trends in gestational diabetes and deliveries	National (2001-2008)
***López-de-Andrés et al. (2017)* [333]**	*European Journal of Internal Medicine*	Renal transplant outcomes in diabetes	Assess incidence and outcomes of renal transplantations in diabetics	National (2002-2013)
***López-Sánchez et al. (2019)* [334]**	*Nefrología*	Hospitalisation in renal replacement therapy	Assess impact of first year of dialysis/transplant on hospitalisations	Madrid (2013-2015)
***Manchon-Walsh et al. (2011)* [335]**	*European Journal of Surgical Oncology*	Rectal cancer	Assess quality variability across hospitals	Catalonia (2005-2007)
***Marco et al. (2011)* [336]**	*Clinical Nutrition*	Malnutrition	Evaluate prevalence and prognostic impact of hospital malnutrition coding	National (2005-2007)
***Marco et al. (2013)* [337]**	*Revista Clínica Española (English Edition)*	Respiratory infections secondary to enteral feeding	Characterise pulmonary complications linked to enteral nutrition	National (2005-2009)
***Marco et al. (2019)* [338]**	*Revista Clínica Española (English Edition)*	Delirium	Describe prevalence and outcomes of delirium in internal medicine	National (2007-2014)
***Martín-Fortea et al. (2015)* [339]**	*Revista de Calidad Asistencial*	Heart failure	Assess improvement in discharge summary completeness	National (2007)
***Martínez-Alés et al. (2020)* [340]**	*General Hospital Psychiatry*	Respiratory failure, infection possible	Analyse use of mechanical ventilation in psychiatric vs general population	National (2000-2015)
***Matesanz-Fernández et al. (2015)* [341]**	*European Journal of Internal Medicine*	Hospital readmissions	Analyse recurrent hospitalisations and causes	Lugo (2000-2012)
***Merchante et al. (2010)* [342]**	*Seguridad Del Paciente*	Hospital safety indicators	Evaluate patient safety events in Madrid hospitals	Madrid (2006)
***Merino et al. (2008)* [343]**	*Enfermedad Tromboembólica Venosa*	Venous thromboembolism	Describe VTE incidence and outcomes	National (1999-2005)
***Montero Ruiz et al. (2021)* [344]**	*Journal of Healthcare Quality Research*	Co-management in ENT	Evaluate effects of shared care on outcomes and resource use	Madrid (2017-2019)
***Moreno López et al. (2020)* [345]**	*Journal of Healthcare Quality Research*	Clinical indicators	Develop indicators to monitor low-value care practices	National (2016)
***Núñez-Gil et al. (2021)* [346]**	*REC: CardioClinics*	Aortic stenosis	Compare outcomes of TAVI vs surgical valve replacement	National (2014-2015)
***Oterino-Moreira et al. (2022)* [347]**	*International Journal of Environmental Research and Public Health*	General hospital mortality prediction	Compare Charlson, Elixhauser, and RCRI using CMBD	Madrid (2017-2021)
***Pastor-García et al. (2020)* [348]**	*Anales de Pediatría*	Congenital heart defects	Describe registry-based congenital anomalies	Valencia (2007-2014)
***Peiró-Pérez et al. (2006)* [349]**	*Gaceta Sanitaria*	Injuries	Describe external causes of injury	National (2002)
***Pérez et al. (1997)* [350]**	*Estudios Geograficos*	General morbidity	Describe national hospital morbidity by age, sex, and region	National (1994)

***Pérez et al. (2014)* [351]**	*Piel*	Dermatological hospitalisations	Describe all dermatology-related hospitalisations	National (2005-2010)
***Piñeiro-Fernández et al. (2024)* [352]**	*Archives of Gerontology and Geriatrics*	Hospitalisations in centenarians	Analyse trends and causes of hospitalisation in patients with more than 100 years	National (2004-2020)
***Quirós López et al. (2012)* [353]**	*Medicina Clínica*	Heart failure	Evaluate prognostic factors in new HF diagnoses	Malaga (1997-2006)
***Reguant et al. (2012)* [354]**	*Revista Española de Anestesiología y Reanimación*	Hip fracture	Identify factors associated with mortality in elderly hip fracture patients	Barcelona (2008)
***Reguant et al. (2013)* [355]**	*Revista Española de Anestesiología y Reanimación*	Hip fracture	Assess risk of bleeding and complications in antiaggregated patients	Barcelona (2008)
***Ribera et al. (2008)* [356]**	*Revista Española de Cardiología (English Edition)*	Cardiac surgery	Compare predictive accuracy of CMBD mortality models vs. clinical registry	Catalonia (2001-2003)
***Rodrigo-Rincón et al. (2016)* [357]**	*Cirugía Española (English Edition)*	Adverse events (surgery)	Evaluate CMBD for surgical risk adjustment	Navarra (2008-2010)
***Rodríguez-Mañero et al. (2017)* [358]**	*International Journal of Cardiology*	Atrial fibrillation	Analyse clinical characteristics and prognosis	Galicia (2013-2015)
***Rodríguez-Mañero et al. (2019)* [359]**	*Revista Portuguesa de Cardiologia*	Atrial fibrillation	Describe prevalence and resource use	Galicia (2013-2015)
***Rodríguez-Sosa et al. (2023)* [360]**	*RMD OPEN*	Gout	Compare comorbidity profiles in women vs men hospitalised with gout	National (2005-2015)
***Romero Ballarín y Oterino de la Fuente (2007)* [361]**	*Revista de Calidad Asistencial*	Thromboprophylaxis	Evaluate adequacy of thromboprophylaxis	Gijón (2005-2006)
***Rué et al. (2001)* [362]**	*Medicina Clínica*	Hospital mortality prediction	Develop probabilistic models for inpatient mortality	Catalonia (1997-1998)
***Rueda Camino et al. (2024)* [363]**	*Revista Clínica Española (English Edition)*	Pulmonary embolism	Describe pregnancy-related PE in Spain	National (2016-2021)
***Sáez López et al. (2015)* [364]**	*Revista Española de Geriatría y Gerontología*	Hip fracture	Evaluate clinical pathway effectiveness	Madrid (2010 vs 2013)
***Sánchez-Gómez et al. (2023)* [365]**	*Acta Otorrinolaringologica (English Edition)*	Airway/tracheostomy care	Describe implementation and outcomes of a dedicated tracheostomy care unit.	Seville (2016-2021)
***Sánchez-Hernández et al. (2016)* [366]**	*Revista Española de Cirugía Ortopédica y Traumatología*	Orthogeriatrics	Evaluate outcomes after implementing a clinical pathway for geriatric hip fracture.	Ávila (2010 vs 2013)
***Scheller-Kreinsen et al. (2013)* [367]**	*The Breast*	Breast cancer	Compare DRG classification and reimbursement for breast surgery across countries.	Catalonia (2008)
***Sendra-Gutiérrez et al. (2018)* [368]**	*Revista de Psiquiatría y Salud Mental*	Suicidal behaviour	Describe hospitalised suicidal behaviour and factors associated with in-hospital mortality.	Madrid (2003-2013)
***Setty et al. (2017)* [369]**	*International Journal of Hygiene and Environmental Health*	Waterborne infection	Assess effects of Water Safety Plan implementation on water quality, compliance, and health outcomes	Catalonia (2003-2015)

***Soria-Aledo et al. (2016)* [370]**	*Cirugía Española (English Edition)*	Postoperative infections	Develop and pilot a core set of general surgery quality indicators	Murcia (2013)
***Tornero Patricio et al. (2017)* [371]**	*Anales de Pediatría (English Edition)*	Respiratory infections (among main causes), asthma, etc.	Assess socioeconomic inequalities in pediatric admissions by postcode	Seville (2014)
***van den Berg et al. (2012)* [372]**	*Archives of Physical Medicine and Rehabilitation*	Spinal cord injury (nontraumatic)	Estimate incidence & causes.	National (1972-2008.)
***Vela et al. (2019)* [373]**	*Gaceta Sanitaria*	Healthcare costs	Quantify healthcare spending by segment.	Catalonia (2014)
***Yetano-Laguna et al. (2006)* [374]**	*Delivered tool for benchmarking across hospitals.*	Quality indicators	Develop 300 automated inpatient quality indicators from CMBD	Basque Region (1995-2005)
***Zamalloa et al. (2019)* [375]**	*Revista Española de Salud Pública*	HIV and other infections included among causes	Describe causes of admission and comorbidities in transgender people	National (2001-2013)
***Barba Martín et al. (2009)* [376]**	*Revista Clinica Espanola*	Internal medicine hospital activity	To describe hospital activity in Internal Medicine departments (2005-2006).	National (2005-2006)
***Zapatero Gaviria et al. (2010)* [377]**	*Medicina Clínica*	Overall mortality causes	Analyse mortality in Internal Medicine services using CMBD.	National (2006)
***Cabeza-Osorio et al. (2022)* [378]**	*Revista Clínica Española*	Internal medicine patients	To analyse characteristics and short-term outcomes of prolonged-stay inpatients.	Madrid (2013-2017)
