# Supplementary material for: Three Decades of Use of the Minimum Basic Data Set in Infectious Disease Research in Spain: A Scoping Review with an Evidence-Mapping Approach
Source: Trop Med Infect Dis. 2026 Feb 20;11(2):61. doi: 10.3390/tropicalmed11020061 (PMC12945255; doi:10.3390/tropicalmed11020061)
Supplement: Supplementary file 1 [file tropicalmed-11-00061-s001.zip › Table S3. Limitations domains and frequency.pdf]

Table S3. Limitations domains and frequency.

Limitation domain (author-reported)	Studies reporting n (%)
**Coding accuracy / misclassification / ICD variability**	114 (31.8%)
**Lack of microbiology / pathogen confirmation**	81 (22.6%)
**Data quality / incomplete capture / heterogeneity across hospitals/regions**	78 (21.7%)
**Limited clinical granularity (severity/treatment/labs/risk factors)**	56 (15.6%)
**Inpatient-only scope (no outpatient/primary care capture)**	48 (13.4%)
**Generalisability / scope restrictions (study-level)**	42 (11.7%)
**Missing variables / residual confounding**	18 (5.0%)
**Vaccination status not available**	14 (3.9%)
**Limited follow-up / post-discharge outcomes**	13 (3.6%)
**Other/unclear non-specific limitation statements**	122 (34.0%)

Studies could report more than one limitation domain; percentages therefore do not sum to 100%. N = 359.
